# Like the new and hate the old: The impact of fiscal decentralization on regional development strategy

**DOI:** 10.1371/journal.pone.0273875

**Published:** 2022-09-09

**Authors:** Min Liu, Feng Gong, Wenjing Song

**Affiliations:** 1 Economics and Management School, Wuhan University, Wuhan, China; 2 School of Public Administration, Zhongnan University of Economics and Law, Wuhan, China; Massey University - Albany Campus: Massey University - Auckland Campus, NEW ZEALAND

## Abstract

This paper evaluates the impact of China’s fiscal decentralization reform, namely the “Province-Managing-County” (PMC) fiscal reform, on local governments’ regional development strategy using county-level data in China covering 2000 to 2013. Surprisingly, after implementing the PMC fiscal reform, local governments will adjust their strategy of supporting zombie firms and attracting new firms, indicating that fiscal decentralization has changed the regional development strategies of local governments. We perform a difference-in-differences (DID) analysis and find that the PMC fiscal reform materially induces an average rise of 0.131 in newly added firms, an average decline of 0.383 in zombie firms, and no significant change in other firms. There is a pronounced substitution effect between zombie firms and newly added firms. We also find evidence supporting this argument: the government’s subsidy, tax treatment, and financial support. Our study provides empirical evidence that local governments’ regional development strategies can be affected by fiscal decentralization.

## 1. Introduction

Local governments not only support “old” insolvent firms to keep them afloat [1], which are widely considered zombie firms, but also give a lot of preferential treatments to attract new firms. However, the regional development strategy of supporting zombie firms is double-edged. Even though keeping zombie firms alive can maintain relatively stable regional development in the short term, it harms the economy in the long term because zombie firms can impede efficient resource allocation [2]. Compared with supporting zombie firms, attracting investment and new firms can promote economic growth, provide new job opportunities, and have no adverse effects. In that case, why do local governments still have an incentive to support zombie firms? Both supporting zombie firms and attracting new firms may be a regional development strategic choice for local governments in the face of limited fiscal resources, but attracting new firms may require more fiscal funds and resources than supporting zombie firms. When the governments’ fiscal capacity changes, they adjust their regional development strategy.

Widely used by many countries around the world, the fiscal decentralization system grants local governments the power to make fiscal support decisions independently. To some extent, fiscal decentralization determines local governments’ fiscal capacity and fiscal autonomy. Second-generation fiscal federalism emphasizes the importance of fiscal decentralization on regional development [3]. Many studies have explored the impacts of fiscal decentralization, such as economic growth [4, 5], public spending [6], et al. However, few studies focus on whether and how fiscal decentralization affects local governments’ regional development strategies. Therefore, this paper is uniquely devoted to bridging this gap. In this paper, we explore the relationship between fiscal decentralization and local governments’ regional development strategy by investigating the effects of large-scale fiscal decentralization reform in China, named the “Province-Managing-County” (PMC) fiscal reform.

**Fig 1:**
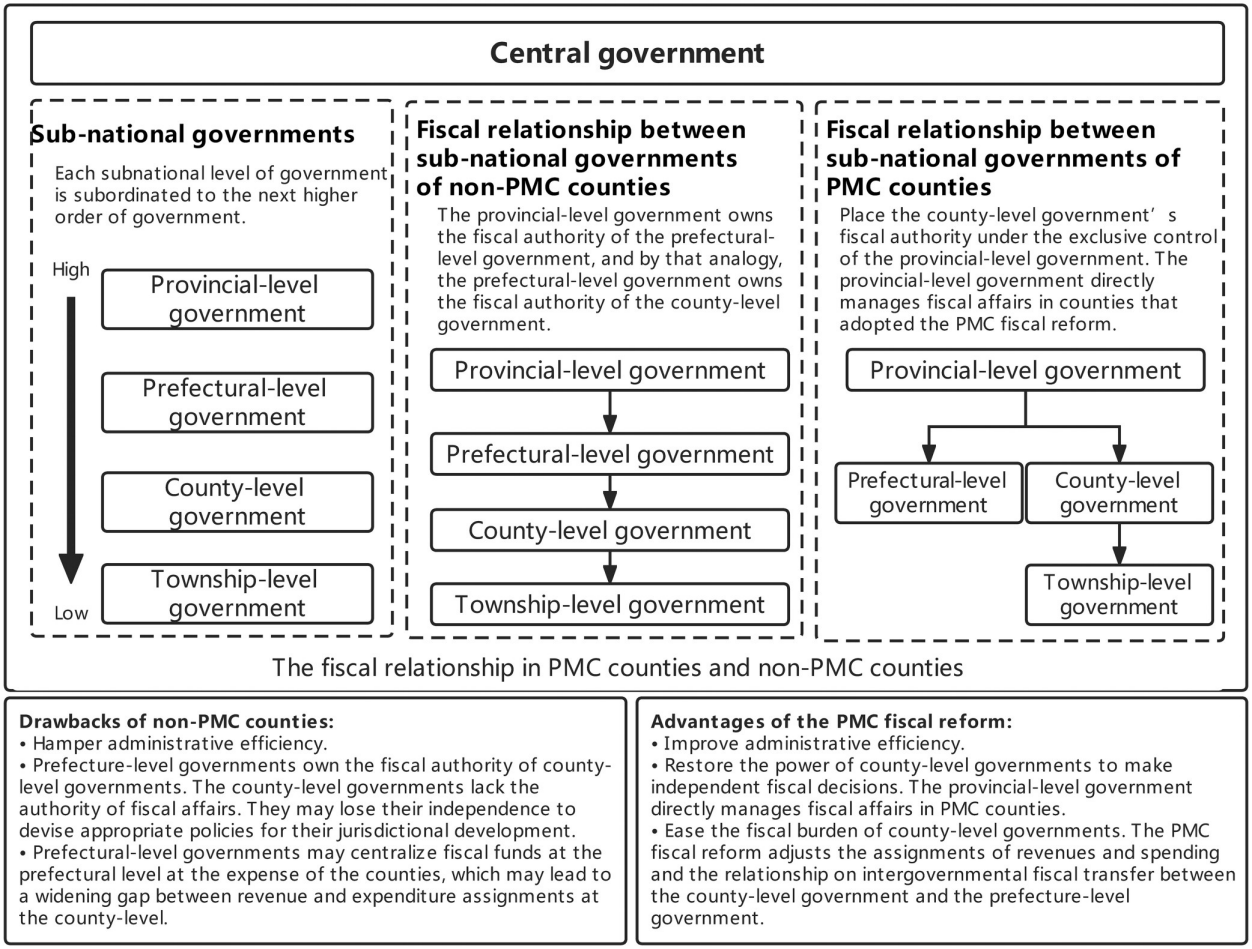
The fiscal relationship between sub-national governments in regions that adopted the PMC fiscal reform (PMC counties) and regions that have not adopted the PMC fiscal reform (non-PMC counties).

Fiscal decentralization refers to transferring fiscal power and responsibility from central to sub-national governments. China’s fiscal system is generally characterized by fiscal decentralization. Sub-national governments in China manage local fiscal affairs on behalf of the central government. There are four layers of sub-national governments in China, from the provincial-level government, the prefectural-level government, and the county-level government to the township-level government. The PMC fiscal reform changes the fiscal relationship between prefectural and county-level governments. Fig 1 shows the fiscal relationship between sub-national governments in regions that adopted the PMC fiscal reform (PMC counties) and regions that have not adopted the PMC fiscal reform (non-PMC counties). Indeed, the PMC fiscal reform promotes fiscal capacity and enhances the fiscal autonomy of PMC counties’ governments [7, 8]. S1 Appendix shows more details about the PMC fiscal reform in China.

Since the PMC fiscal reform is implemented step-by-step, we assess the causal relationship between the PMC fiscal reform and local governments’ regional development strategy in a difference-in-differences (DID) setting. We first document that fiscal decentralization notably affects local governments’ regional development strategies for zombie firms and new firms. Concretely speaking, the PMC fiscal reform has a positive impact on the newly added firms, a negative impact on the zombie firms, and no impact on other firms (firms excluding zombie firms and newly added firms) using county-level data from 2000 to 2013. It indicates that the PMC fiscal reform increases local governments’ willingness to attract investment but decreases their desire to adopt the strategy of keeping insolvent zombie firms afloat.

However, one possibility is that one may think the results are erroneous since the PMC counties are not randomly selected, i.e., a county can be chosen as the PMC county for its specific natural characteristics. To address this concern, following Li et al. [9], we control for eight key determinants that influence a county chosen as a PMC county to eliminate the effects of sample selection and interact these key determinants with the year dummies to control for the time-varying effects. Also, we include treatment-specific linear time trends in our analysis to control for time trends between the PMC counties and non-PMC counties. Moreover, we use three different methods to identify zombie firms and conduct separate analyses to confirm the robustness of the impacts. Finally, we also try to use a series of robustness tests and a placebo test. We verify that our findings hold up under various conditions and specifications. The convincing evidence adds weight to our main result.

So how to explain our main findings? One mechanism that may generate such variation is that the PMC fiscal reform changes governments’ strategy for supporting zombie firms and attracting new firms. There is a pronounced substitution effect between zombie firms and newly added firms. The PMC fiscal reform may induce local governments’ preference for newly added firms and reduce local governments’ preference for zombie firms. We investigate three possible channels to prove the mechanism. We view this analysis component as a preliminary exploration of each explanation needing deeper investigation. Three kinds of evidence come from examining the changes in local government subsidy, tax burden, and financial support between zombie firms and newly added firms resulting from the PMC fiscal reform.

To the best of our knowledge, our study is one of the first pieces of empirical analysis to establish the relationship between fiscal decentralization and local governments’ regional development strategies. We extend the research by exploring the effects of fiscal decentralization and retest the theory of second-generation fiscal federalism [3]. Our study fully demonstrates that regional development strategies can be affected by fiscal decentralization by showing that the PMC fiscal reform changes the local government’s development strategy for new firms and zombie firms.

Also, our study proves that government intervention is an important cause for the formation and existence of zombie firms in China from a reverse perspective. Existing studies have shown that government intervention [10], such as subsidies [11] or resource support [12], has been shown as the main cause of zombie firms in China. In this paper, we show that when governments’ support for zombie firms decreases, the number of zombie firms decreases. Our study demonstrates the importance of government intervention to zombie firms from another research perspective.

The remainder of the paper proceeds as follows. Section 2 describes the theoretical framework and literature review. Section 3 describes the methodology. Section 4 provides the empirical results. Section 5 provides the mechanism. Section 6 concludes the paper.

## 2. Theoretical framework and literature review

### 2.1 Theoretical framework: The PMC fiscal reform and regional development strategy

#### 2.1.1 The strategy of supporting zombie firms

Zombie firms refer to low profitability firms that cannot afford debt-servicing costs from current profits for an extended period [13]. They keep alive by various forms of external financial support, such as “evergreen” loans from banks or subsidies from the government [14], rather than standing on their own feet. Zombie firms have sparked global concern as they drag on economic growth by impeding efficient resource allocation [2]. Studies in developed countries show that evergreen lending from banks and low-interest rates [13] are the leading causes of zombie firms. However, government intervention in forming zombie firms attracts more and more attention in developed and developing countries. Studies conducted by Chang et al. [12] and Shen & Chen [15] reveal that fiscal support from the local government is another contributor to the formation of zombie firms in China.

Local governments support zombie firms as a strategy to stabilize regional development. Even though zombie firms are harmful to the economy mainly by occupying various kinds of resources that could utilize by normal firms, which may impose a cost on the economy [2], they can also absorb employment, generate GDP, and generate fiscal revenues to maintain economic stability and job security. If zombie firms lack support from local governments, they will face the risk of bankruptcy and withdrawal from the market [16]. It will create a lot of unemployed people in the region [1] and reduce the region’s GDP and tax revenue [1, 12].

#### 2.1.2 The strategy of attracting new firms

Local governments treat attracting investment and new firms as a strategy to promote regional development. Therefore, it is essential to create a supportive environment for investment and new firms [17]. Attracting investment and new firms benefits the region a lot. It can promote local economic development, optimize the regional industrial structure, and bring capital, technology, products, and management methods. China’s unique “economic decentralization and political centralization” system prompts local governments to compete in attracting investment and new firms to develop the local economy. Government behaviors have a significant impact on economic growth [18]. Many empirical studies based on Chinese data show that various forms of competition in attracting investment and new firms among local governments are fierce [19]. Local governments usually want to attract more new firms to settle down, increase regional GDP, increase fiscal revenues, and create more jobs. If the local government fails to attract investment, it will adversely affect the local economy and political performance.

However, attracting investment and new firms requires the support of the local government’s fiscal resources and fiscal autonomy. Local governments usually offer preferential treatments to attract more investments and new firms, which can bring direct economic benefits to firms and lay the groundwork for developing new firms after they settle down. Preferential treatments include direct subsidies, land price subsidies [20, 21], and tax credit [22, 23], and groundwork consists of a sound business environment and excellent infrastructure [24]. Therefore, all the measures of local governments to attract investment and new firms need the support of fiscal funds. However, the fiscal resources can be used to attract investment and new firms are limited. Although attracting investment and new firms can significantly promote regional development, the local government’s investment attraction behavior is also restricted.

#### 2.1.3 Impacts of the PMC fiscal reform on regional development strategy

The intention and motivation of local governments to support zombie firms and attract new firms are different. Local governments support zombie firms to avoid layoffs, fiscal revenue loss, and economic depression [25, 26] while attracting new firms to promote economic growth and provide new job chances. Since fiscal funds and fiscal autonomy of local governments are finite, the local governments’ fiscal resources to support zombie firms and attract new firms should match their local conditions. Based on the analysis of regional development conditions, local governments will choose the suitable fiscal fund allocation method between supporting zombie firms and attracting new firms.

**Fig 2:**
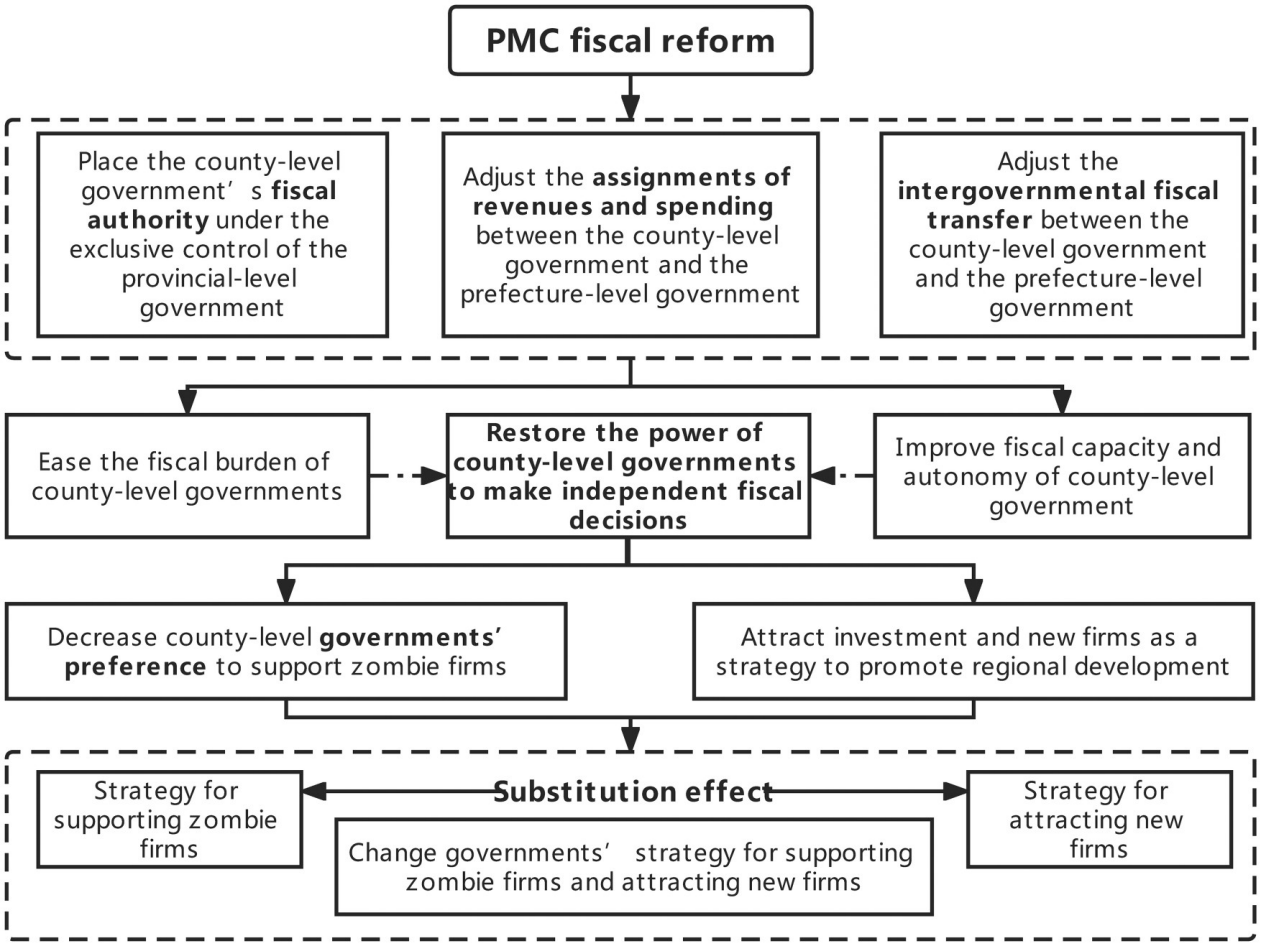
The conceptual framework.

The hierarchical organization of government is closely linked with regional development [27, 28]. The PMC fiscal reform is a reform that flattens government hierarchies [9]. A study by Li et al. [9] shows that the PMC fiscal reform not only improves the administrative efficiency of governments but also eases the fiscal burden and enhances the fiscal capacity of county-level governments. The improvement of fiscal autonomy of county-level government caused by the PMC fiscal reform makes previous choices on fiscal fund allocation method between supporting zombie firms and attracting new firms no longer suitable. Local governments’ strategy of supporting zombie firms and attracting new firms may change. Fig 2 is the conceptual framework.

On the one hand, the PMC fiscal reform eases the fiscal burden and increases the fiscal capacity of county-level governments [9], which makes the tax revenue loss caused by zombie firms’ bankruptcy have less influence on the fiscal revenue of county-level governments. This, in turn, may decrease county-level governments’ preference to support zombie firms.

On the other hand, the PMC fiscal reform enhances the fiscal capacity of county-level governments, which may affect the regional development strategy of county-level governments. Before implementing the PMC fiscal reform, the arrangements of county-level governments’ fiscal budgets should be audited by the prefecture-level governments. Therefore, the fiscal funding arrangements of counties are often more in line with the willingness of prefecture-level governments rather than the county-level government’s own [9, 29]. Indeed, the lack of fiscal autonomy limits county-level governments’ ability to develop their regional economy. Thus, the local governments have adopted a stop-gap measure of keeping zombie firms alive to maintain relatively stable regional development since zombie firms can provide jobs and maintain local economic development [1, 12]. Implementing the PMC fiscal reform has made the county-level governments richer and grants county-level governments more fiscal autonomy. Supporting unprofitable zombie firms to maintain relatively stable regional development is no longer a suitable strategy for local governments. They have the ability and autonomy to take other measures to promote regional development better and decrease the fiscal support for zombie firms. Since investment attraction can boost jobs [30], impetus economic growth [31], et al., they may prefer to attract investment. As a result, governments’ preference for zombie firms may decrease, and governments’ preference for attracting new firms may increase.

On these bases, we argue that implementing the PMC fiscal reform may reduce local governments’ preference for zombie firms and increase local governments’ preference for attracting new firms. This, in turn, may reduce regional zombie firms that cannot survive independently and grow regional new firms. It will result in a pronounced substitution effect between zombie firms and new firms.

### 2.2 Literature review

This paper first contributes to the literature evaluating fiscal decentralization’s impact. The majority of research in this area has concentrated on the effects on economic growth [4, 5], local tax enforcement [32], government quality [33], the size of government [34], public spending [6], public debt [35], intergovernmental fiscal relations [36], students education outcomes [37], intra-provincial inequality [38], public service [39], and so on. Despite this evidence, little effort has been devoted to detecting the effect of fiscal decentralization on regional development strategy. Our work bridges the gap by assessing the impacts of fiscal decentralization on government’s development strategy from the perspective of new firms and zombie firms. Also, our study departs from the issues by rigorously addressing the causal identification problem of troubled fiscal decentralization measurement using the DID method.

Second, our analysis contributes to the separate branch of burgeoning literature looking at the effects of China’s “Province-Managing-County” (PMC) fiscal reform (i.e., land supply [40], government spending patterns [7]). Many studies have shown that the PMC fiscal reform can promote economic growth in China [40, 41]. However, there is still a lack of research detecting the effects of the PMC fiscal reform on other aspects of economic development in China. Our study shows that the PMC fiscal reform can increase the newly added firms and decrease zombie firms, promoting high-quality economic growth in China.

Third, our analysis also extends the literature about zombie firms. The research on zombie firms is mainly divided into three branches. The first branch of literature focuses on the identification and causes of zombie firms. Studies conducted by Caballero et al. [42] and Fukuda and Nakamura [43] are the most typical. The second branch of literature looks at the impacts of zombie firms. The most noticeable impact of zombie firms is that they can impede efficient resource allocation [2] and thus do harm to the development of the economy [42, 44–46]. The third branch of literature focuses on reducing zombie firms [10, 47]. A growing number of literature focus on the effect of government behavior and the government system on zombie firms, such as government subsidies [11], government resource support [12], and political relationships [48]. Cai et al. [49] show that the PMC fiscal reform can inhibit the formation of zombie firms, proving that the local governments’ fiscal stress contributes to the formation of zombie firms. However, it does not explore why local government subsidizes zombie firms before the PMC fiscal reform and then reduces the support for zombie firms after the PMC fiscal reform. Our study links fiscal decentralization and the changes in local governments’ strategy for zombie firms and shows the in-depth reason for the local governments’ strategy changes between the zombie firms and new firms.

Finally, this paper adds to the literature on investment attraction. Some literature focus on the influence factors of investment attraction, especially for the foreign direct investment (FDI), such as environmental regulation [50], corruption [51], financial [52], fiscal transparency [53], fiscal decentralization [54], subsidy [55], regional social context [56], and so on. Bailey [57] use the meta-analysis method to find the relationship between institutional factors and host country FDI, and he finds that political stability, democracy, and the rule of law attract FDI, and corruption, tax rates, and cultural distance deter it. Janeba [58] studies the influence factor of the host governments’ subsidy willingness for FDI. Tian [59] compares the efficiency of tax rate reduction policy and investment cost subsidy policy for the host government to attract FDI. Some literature focus on the impacts of the inflow of FDI, such as entrepreneurial activities [60], country welfare [61], regional economic growth [62], pollution [63], et al. However, a lack of research studies on the reasons why local governments change their preference for investment attraction. We provide the extension to motivate and guide future research on the channels linking fiscal decentralization and local governments’ investment attraction action.

## 3. Methodology

To assess the effect of the PMC fiscal reform on regional development strategy, we gather data on the timing and counties of the PMC fiscal reforms, firm-level datasets, and county-level characteristics over the period 2000–2013. This section presents the data and describes the econometric methods.

### 3.1 Data

This paper’s county-level and firm-level data are collected from multiple sources. We construct a county-level data set to describe county-level socioeconomic conditions. County-level data are collected from China County Statistical Yearbook. Each county’s average geographic slope and altitude are constructed using ArcGIS software.

Firm-level data comes from the China Annual Surveys of Industrial Firms (CASIF), which is conducted by China’s National Bureau of Statistics (NBS) and is widely used in many studies [64–68]. For example, Zhao et al. [64] analyze the impact of China’s development zone plans on haze pollution by using CASIF data set from 2003 to 2013. The CASIF contains basic information about all state-owned firms and the above-scale non-state firms (above-scale non-state firms refer to non-state firms with above 5 million RMB in revenue before 2011 and 20 million RMB after 2011), such as name, firm ID code, address, two-digit industrial classification code, four-digit industrial classification code, and so on. Due to the availability of CASIF, like other studies used the CASIF, the sample period of our study is from 2000 to 2013.

Following Cai and Liu [69], we exclude three kinds of observations from the original CASIF data set to get a clean sample, including observations whose critical parameters are missing, whose data do not meet accounting standards, and whose employees are less than 10. Then, we use unique firm ID codes to link firms in CASIF over time. To the firms who change firm ID, we use their name, four-digit industry code, address, etc., to link them [70]. We also use a four-digit industrial classification code to differentiate industries of firms. Each firm in the CASIF is classified into an industry according to the industrial classification code from the Chinese Industrial Classification for National Economic Activities (CICNEA). The first CICNEA was published in 1984 and was revised in 1994, 2002, 2011, and 2017. In our sample period, we use the 2011 revision CICNEA to adjust all the firms’ four-digit industrial classification codes to make our analysis more accurate.

Following studies by Li et al. [9] and Huang et al. [7], we eliminate the following counties in our study. First, counties in the four centrally administered municipalities (Beijing, Shanghai, Chongqing, and Tianjin). Second, counties in Hainan and Zhejiang provinces adopted the PMC fiscal reform in the late 1980s, and counties in Tibet due to data deficiency. Third, counties with administrative affiliations change. To identify the effect of the PMC fiscal reform more precisely, we also excluded counties that adopted the Reinforcing Counties by Grating More Powers (RCGP) reform. Thus, the sample for empirical analysis consists of a panel of 977 counties over the 2000–2013 period. Monetary units in the data set are adjusted using provincial price deflators.

### 3.2 Estimation strategy

#### 3.2.1 DID method

We assess the causal relationship between the PMC fiscal reform and regional development strategy in a DID setting. The main benefit of using the DID approach is that it allows us to control for time-invariant, county-specific omitted variables, time-varying county development trends, and nationwide shocks to the variables of interest.

One possibility is that one may think the results have some deviation since the PMC counties are not randomly selected. To address this concern and improve the identification, following Li et al. [9], we control for eight key determinants that affect the selection of PMC counties. To control for the time effects, the eight key determinants have interacted with the year dummies *T*_*t*_. Beyond that, we also include treatment-specific linear time trends to control for the time trends between the PMC counties and non-PMC counties. We estimate the equation based on the following augmented regression set-up:

$$Y_{it}=\alpha_{0}+\alpha_{1}Reform_{it}+\beta_{i}Treat_{i}\cdot t+{\left(M\times T_{t}\right)}^{\prime }\theta +X_{it}+C_{i}+T_{t}+\varepsilon_{it}$$ (1)

In Eq (1), *Y*_*it*_ is the number of firms in county *i* in year *t*, including newly added firms, zombie firms, and other firms (firms excluding newly added firms and zombie firms). *Treat*_*i*_ t is treatment-specific linear time trends to control between PMC and non-PMC counties. *M* is a vector of eight key determinants in the selection of PMC counties, including the county-level city, the NPC (national poverty-stricken county), the food-producing county, the province boundary county, the geographic slope, the altitude, the urbanization rate in 2000, and the fiscal gap in 1999 [9]. (*M* × *T*_*t*_)′ is used to control for the chronological evolution of the outcome variables whose correlation with PMC is caused by the endogenous pattern of PMC selection [9]. *X*_*it*_ is a set of time-varying county-level control variables including PGDP, population density, regional finance development, and industrial structure. *C*_*i*_ and *T*_*t*_ are vectors of county and year dummy variables that account for county and year fixed effects. *C*_*i*_ is used to control for time-invariant, unobserved county characteristics that affect outcome variables and *T*_*t*_ is used to control for nationwide shocks and trends that affect outcome variables in counties over time. *ε*_*it*_ is the error term. We estimate Eq (1), allowing for county-level clustering of the errors.

The variable of interest is *Reform*_*it*_, which is a dummy variable equaling one in the years after county *i* started the PMC fiscal reform and zero otherwise. The coefficient, *α*_1_, therefore indicates the impacts of the PMC fiscal reform on newly added firms, zombie firms, and other firms. A positive and significant *α*_1_ suggests that the PMC fiscal reform exerts a positive effect on counties’ firms, while a negative and significant one indicates that the PMC fiscal reform lowers counties’ firms. In total, we have data for 977 counties over 14 years, including 323 PMC counties and 654 control groups, so the 13678 county-year observations serve as the basis for much of our analysis.

#### 3.2.2 Zombie firm and newly added firms identification methods

The generally accepted approaches to identifying zombie firms are the CHK method and the FN-CHK method based on Japanese zombie firms’ research. In a study by Caballero et al. [42], zombie firms are defined as firms whose hypothetical risk-free interest payments are higher than interest payments. They detect the zombie firms by finding whether they received subsidized credit from banks, afterward named the CHK method.

Fukuda and Nakamura [44] developed a new method called the FN-CHK method based on the CHK method. FN-CHK method has introduced two additional criteria for zombie firms. The first one is the ‘profitability criterion’. If a firm’s earnings before interest and taxes (EBIT) are higher than the hypothetical risk-free interest payments, the firm will be excluded from the zombie firms. The second one is the “evergreen lending criterion”. If the firm’s EBIT for a specific year was less than the hypothetical risk-free interest payments, the total external debt of the previous year is higher than half of its total assets, and the borrowing of that year has increased, the firm will be defined as a zombie firm. More details about the zombie firms identification method are shown in S1 Appendix.

Even though the FN-CHK method offers a more accurate picture of zombie firms, it still exists a drawback in that some firms are identified as zombie firms only in one year of the entire study period. This is inconsistent with the understanding of zombie firms. To address the shortcoming of the FN-CHK method, Nie et al. [71] from the National Academy of Development and Strategy of Renmin University of China propose the Nie method: when a firm is identified as a zombie firm by the FN-CHK method both in a specific year and the year before that year, the Nie method identifies it as a zombie firm.

Government fiscal support is an important contributor to the formation of zombie firms in China [12]. Some unhealthy firms may be mistakenly identified as healthy firms because they receive government fiscal support. To isolate the impact, Tan et al. [46] proposed the Tan method to identify zombie firms according to the actual situation in China. In this method, the firm profit is replaced by firm operating profit based on the Nie method, considering that firm profit may include government fiscal support or other non-recurring profit and loss. Due to the strength and weaknesses of these methods, we use three methods to identify zombie firms: the Nie method, the Tan method, and the FN-CHK method in our study.

Newly added firms are identified by the time they are established. For example, a firm established in 2003 will be recognized as a newly added firm in 2003.

## 4. Empirical result

### 4.1 Descriptive statistical analysis

Table 1 shows the definitions and descriptions of variables in our study. We quantified the variables in PMC and non-PMC counties, such as newly added firms, zombie firms, other firms, PGDP, population density, regional finance development, et al. We find that the average number of newly added firms in PMC counties is larger than in non-PMC counties, and so are zombie firms and other firms. We also find that PMC counties have smaller fiscal gaps than non-PMC counties, which indicates that the PMC counties have lower fiscal pressure than non-PMC counties. It is consistent with our theoretical framework.

**Table 1:** Descriptive statistics.

Variable	Description	PMC counties			Non-PMC counties			All counties		
		Mean	S.D.	Obs.	Mean	S.D.	Obs.	Mean	S.D.	Obs.
**Newly added firms**	One plus the number of newly added firms in counties (natural log)	0.610	0.802	4522	0.533	0.820	9156	0.558	0.815	13678
**Zombie1**	One plus the number of zombie firms identified by the Nie method in counties (natural log)	1.108	0.899	4522	1.075	0.930	9156	1.086	0.920	13678
**Zombie2**	One plus the number of zombie firms identified by the Tan method in counties (natural log)	1.101	0.898	4522	1.075	0.930	9156	1.083	0.919	13678
**Zombie3**	One plus the number of zombie firms identified by the FN-CHK method in counties (natural log)	1.502	0.960	4522	1.460	1.031	9156	1.474	1.008	13678
**Other firms1**	One plus the number of other firms in counties (natural log, firms excluding zombie firms in Nie method and newly added firms)	2.968	1.128	4522	2.724	1.387	9156	2.805	1.312	13678
**Other firms2**	One plus the number of other firms in counties (natural log, firms excluding zombie firms in Tan method and newly added firms)	2.970	1.127	4522	2.722	1.388	9156	2.804	1.312	13678
**Other firms3**	One plus the number of other firms in counties (natural log, firms excluding zombie firms in FN-CHK method and newly added firms)	2.846	1.173	4522	2.601	1.416	9156	2.682	1.345	13678
**PGDP**	Gross Domestic Product (GDP) per capita (¥, natural log)	8.912	0.837	4519	9.201	0.935	9138	9.105	0.914	13657
**Pop density**	Population density (persons per square kilometer, natural log)	5.255	1.150	4522	4.339	1.761	9156	4.642	1.642	13678
**Regional finance development**	Total loan balance of financial institutions at the end of year (ten thousand ¥, natural log)	11.375	0.970	4120	11.248	1.335	8914	11.288	1.233	13034
**Industrial structure**	Proportion of the GDP of the secondary and tertiary industries (%)	72.967	11.983	4349	71.048	14.734	8890	71.678	13.919	13239
**County-level city**	= 1 if a county is a county-level city; = 0 otherwise	0.080	0.272	4522	0.136	0.343	9156	0.118	0.322	13678
**NPC**	= 1 if a county is a national poverty-striken county; = 0 otherwise	0.427	0.495	4522	0.327	0.469	9156	0.360	0.480	13678
**Food-producing county**	= 1 if a county is a national food or cotton production county; = 0 otherwise	0.245	0.430	4522	0.180	0.385	9156	0.202	0.401	13678
**Provboundary county**	= 1 if a county’s boundary (at least part of it) is overlapped with its provincial boundary; = 0 otherwise	0.464	0.499	4522	0.343	0.475	9156	0.383	0.486	13678
**Geographic slope**	Average county geographic slope (degrees) in year 1999	8.852	6.292	4522	10.089	7.675	9156	9.680	7.270	13678
**Altitude**	County seat’s altitude (km) in year 1999	0.721	0.820	4522	1.078	1.030	9156	0.960	0.980	13678
**Urbanization rate**	Percentage of non-agricultural population in the total population in year 2000	12.397	6.513	4522	17.852	13.802	9156	16.048	12.170	13678
**Fiscal gap**	Ratio of fiscal expenditure to fiscal revenue in year 1999	2.750	2.180	4522	3.338	3.565	9156	3.144	3.186	13678

### 4.2 The impacts of the PMC fiscal reform on regional development strategy

We first explore the impacts of the PMC fiscal reform on regional development strategy. We use the number of newly added firms as a proxy for county-level governments’ investment attraction activities and the number of zombie firms for county-level governments’ maintaining economic stability activities. We control county and year fixed effect, treatment-specific linear time trends, eight key PMC county selection variables interacted with year dummies, and additional control variables, including PGDP, population density, regional finance development, and industrial structure.

Column (1) of Table 2 reports the impacts of the PMC fiscal reform on county-level governments’ investment attraction activities. The result shows that the PMC fiscal reform has a significant positive impact on the number of newly added firms. Column (2) indicates that the PMC fiscal reform caused a significant negative effect on zombie firms by using the Nie method. The results presented so far cast doubt on that the apparent impact of the PMC fiscal reform on newly added firms and zombie firms may also happen to other firms. To address the concern, column (3) further analyzes the impacts of the PMC fiscal reform on other firms. The insignificant results indicate that the estimate of the impacts of the PMC fiscal reform has no significant effect on the number of other firms.

**Table 2:** The impact of the PMC fiscal reform on regional development strategy.

	(1)	(2)	(3)	(4)	(5)	(6)	(7)
**PMC fiscal reform**	0.131***	-0.149***	0.022	-0.145***	0.020	-0.089**	0.033
	(0.048)	(0.045)	(0.036)	(0.046)	(0.036)	(0.043)	(0.037)
**Year fixed effect**	Yes	Yes	Yes	Yes	Yes	Yes	Yes
**County fixed effect**	Yes	Yes	Yes	Yes	Yes	Yes	Yes
**Control variables**	Yes	Yes	Yes	Yes	Yes	Yes	Yes
**8 key determinants ×Year**	Yes	Yes	Yes	Yes	Yes	Yes	Yes
**Treatment trend**	Yes	Yes	Yes	Yes	Yes	Yes	Yes
**Year coverage**	2000–2013	2000–2013	2000–2013	2000–2013	2000–2013	2000–2013	2000–2013
**Adjusted R-squared**	0.466	0.676	0.875	0.678	0.875	0.714	0.876
**Obs**.	12764	12764	12764	12764	12764	12764	12764

Notes: Column (1) report the effect of the PMC fiscal reform on the number of newly added firms plus one. Columns (2), (4), and (6) report the effect of the PMC fiscal reform on the number of zombie firms plus one by using the Nie method, Tan method, and FN-CHK method. Columns (3), (5), and (7) report the effect of the PMC fiscal reform on the number of other firms plus one by using the Nie method, Tan method, and FN-CHK method.

*, **, and *** indicate statistical significance at the 10%, 5% and 1% levels respectively.

Next, we change the zombie firm identification method into the Tan and FN-CHK methods to check the identification’s robustness. Columns (4) and (6) report the impact of the PMC fiscal reform on zombie firms by using the Tan method and the FN-CHK method, and columns (5) and (7) show the impact of the PMC fiscal reform on other firms by using Tan method and the FN-CHK method. Results in columns (4) to (7) indicate that the negative impacts of the PMC fiscal reform on zombie firms and the insignificant impacts of the PMC fiscal reform on other firms are robust.

The results in Table 2 show that the PMC fiscal reform has a significant positive effect on the number of newly added firms and a significant negative impact on zombie firms. Further analysis indicates that the PMC fiscal reform has no significant impact on the number of other firms. These results suggest that the PMC fiscal reform has changed county-level governments’ regional development strategy, mainly reflected in the fact that local governments increased the willingness to attract investment and decreased the desire to adopt the approach of keeping insolvent zombie firms afloat.

### 4.3 Robustness checks: Sample of PMC counties

We restrict the estimation sample to the PMC counties. The identification relies on comparing the PMC fiscal reform’s effects between early-adopting counties and later-adopting ones [72]. Column (1) in Table 3 reports the impact of the PMC fiscal reform on the number of newly added firms plus one. Columns (2), (4), and (6) report the effect of the PMC fiscal reform on the number of zombie firms plus one by using the Nie method, Tan method, and FN-CHK method. Columns (3), (5), and (7) report the effect of the PMC fiscal reform on the number of other firms plus one by using the Nie method, Tan method, and FN-CHK method. In Table 3, the PMC fiscal reform dummy enters positive significance at the 10% level in column (1), negative significance in columns (2), (4), and (6), and insignificant in columns (3), (5) and (7). Results in Table 3 show that the PMC fiscal reform has a significant positive effect on the number of newly added firms and a significant negative effect on zombie firms, indicating the estimations of the PMC fiscal reform’s effect are robust.

**Table 3:** Robustness checks: Sample of PMC counties.

	(1)	(2)	(3)	(4)	(5)	(6)	(7)
**PMC fiscal reform**	0.127**	-0.160***	-0.002	-0.179***	-0.003	-0.143***	0.020
	(0.053)	(0.054)	(0.040)	(0.058)	(0.041)	(0.053)	(0.041)
**Year fixed effect**	Yes	Yes	Yes	Yes	Yes	Yes	Yes
**County fixed effect**	Yes	Yes	Yes	Yes	Yes	Yes	Yes
**Control variables**	Yes	Yes	Yes	Yes	Yes	Yes	Yes
**8 key determinants ×Year**	Yes	Yes	Yes	Yes	Yes	Yes	Yes
**Treatment trend**	Yes	Yes	Yes	Yes	Yes	Yes	Yes
**Year coverage**	2000–2013	2000–2013	2000–2013	2000–2013	2000–2013	2000–2013	2000–2013
**Adjusted R-squared**	0.416	0.691	0.830	0.684	0.830	0.710	0.835
**Obs**.	3994	3994	3994	3994	3994	3994	3994

Notes: Column (1) report the effect of the PMC fiscal reform on the number of newly added firms plus one. Columns (2), (4), and (6) report the effect of the PMC fiscal reform on the number of zombie firms plus one by using the Nie method, Tan method, and FN-CHK method. Columns (3),(5), and (7) report the effect of the PMC fiscal reform on the number of other firms plus one by using the Nie method, Tan method, and FN-CHK method.

*, **, and *** indicate statistical significance at the 10%, 5% and 1% levels respectively.

### 4.4 Robustness checks: PSM-DID

Matching the control and treatment groups can effectively solve the problem of sample heterogeneity and selection bias. To ensure that the research results are more reasonable and reliable [73, 74], we use the PSM method to match a suitable control group for the treatment group using kennel matching [73]. Table 4 reports the results. The PMC fiscal reform dummy enters positive significance at the 10% level in column (1), negative significance in columns (2), (4), and (6), and insignificant in columns (3), (5), and (7). The results are similar to Tables 2 and 3, showing that the PMC fiscal reform’s effect estimation is robust.

**Table 4:** Robustness checks: PSM-DID.

	(1)	(2)	(3)	(4)	(5)	(6)	(7)
**PMC fiscal reform**	0.151***	-0.131***	-0.003	-0.131***	-0.003	-0.097**	0.013
	(0.049)	(0.045)	(0.036)	(0.045)	(0.036)	(0.044)	(0.037)
**Year fixed effect**	Yes	Yes	Yes	Yes	Yes	Yes	Yes
**County fixed effect**	Yes	Yes	Yes	Yes	Yes	Yes	Yes
**Control variables**	Yes	Yes	Yes	Yes	Yes	Yes	Yes
**8 key determinants ×Year**	Yes	Yes	Yes	Yes	Yes	Yes	Yes
**Treatment trend**	Yes	Yes	Yes	Yes	Yes	Yes	Yes
**Year coverage**	2000–2013	2000–2013	2000–2013	2000–2013	2000–2013	2000–2013	2000–2013
**Adjusted R-squared**	0.457	0.689	0.877	0.693	0.876	0.723	0.875
**Obs**.	11405	11405	11405	11405	11405	11405	11405

Notes: Column (1) report the effect of the PMC fiscal reform on the number of newly added firms plus one. Columns (2), (4), and (6) report the effect of the PMC fiscal reform on the number of zombie firms plus one by using the Nie method, Tan method, and FN-CHK method. Columns (3), (5), and (7) report the effect of the PMC fiscal reform on the number of other firms plus one by using the Nie method, Tan method, and FN-CHK method.

*, **, and *** indicate statistical significance at the 10%, 5% and 1% levels respectively.

### 4.5 Dynamics of the PMC fiscal reform and regional development strategy

Then we examine the dynamics of the relationship between the PMC fiscal reform and regional development strategy. Following Beck et al. [75], a series of dummy variables in the baseline regression are included to delineate the year-by-year effects of the PMC fiscal reform on firms:

$$Y_{it}=\alpha_{0}+\alpha_{1}T_{it}^{-7}+\alpha_{2}T_{it}^{-6}+\cdot \cdot +\alpha_{1}T_{it}^{+4}+\beta_{i}Treat_{i}\cdot \text{t}+{\left(M\times T_{t}\right)}^{\prime }\theta +{\text{X}}_{it}+C_{i}+T_{t}+\varepsilon_{it}$$ (2)

**Fig 3:**
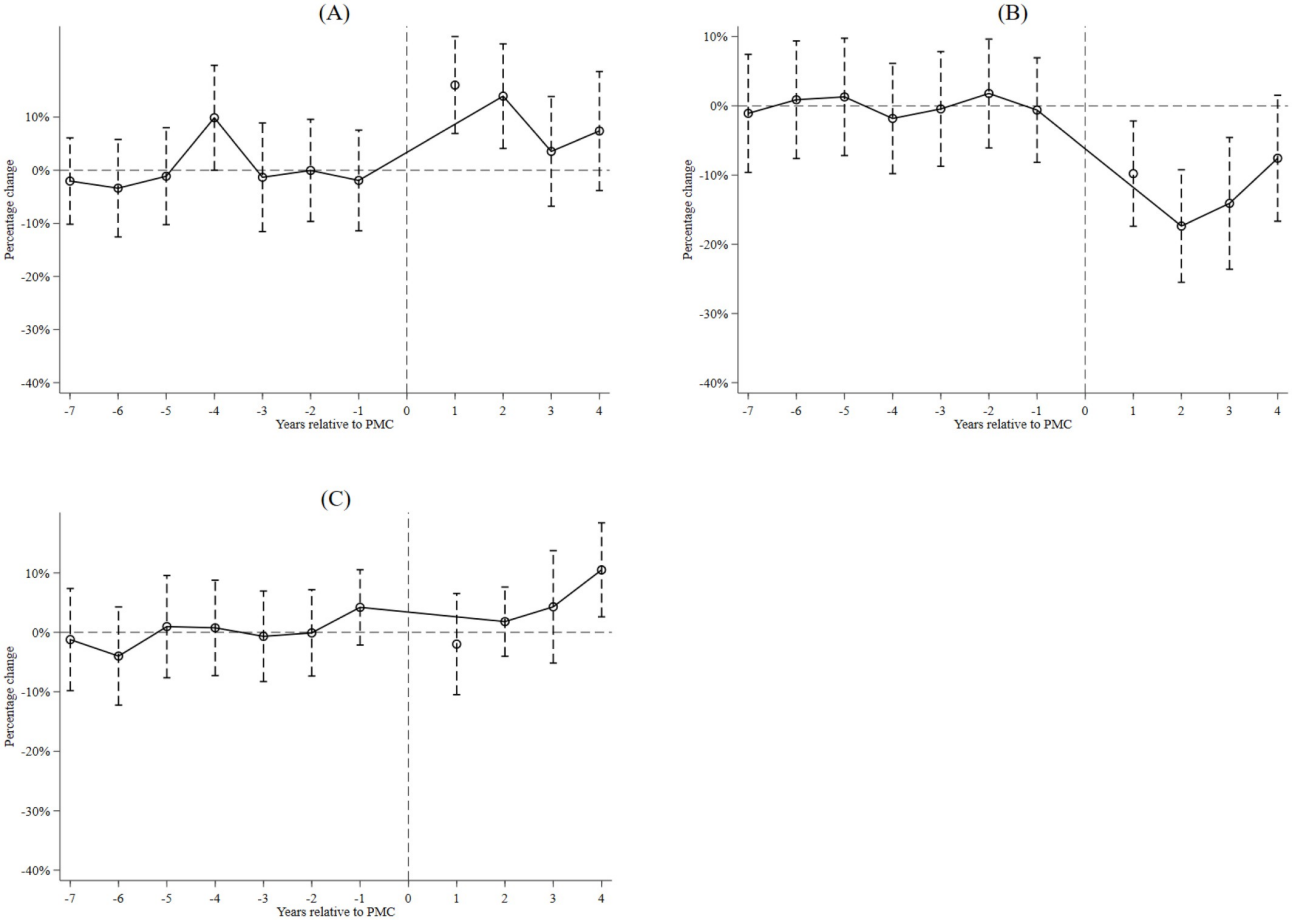
Dynamics of the PMC fiscal reform and regional development strategy. Notes: Panel A is the newly added firms, Panel B is the zombie firms, and Panel C is the other firms. The zombie firm identification method is the Nie method. We use an 11-year window spanning seven years before the PMC fiscal reform until four years after the PMC fiscal reform. The dashed lines represent 95% confidence intervals.

“*T*’s” are the PMC fiscal reform dummy variables and equal zero except as follows: *T*^*-m*^ equals one for counties in the *m*th year before adopting the PMC fiscal reform while *T*^*+m*^ equals one for counties in the *m*th year after adopting the PMC fiscal reform. *Y*_*it*_ is the amount of newly added firms, zombie firms, and other firms in county *i* in year *t*. To estimate the dynamic effects of the PMC fiscal reform on newly added firms, zombie firms, and other firms relative to the year the PMC fiscal reform was implemented, the year the PMC fiscal reform was launched has been excluded. *C*_*i*_ is county fixed effects and *T*_*t*_ is year fixed effects. In Eq (2), $T_{it}^{-7}$ equals one for all years that are seven years before the year the county adopted the PMC fiscal reform, $T_{it}^{+4}$ equals one for four years after the county adopted the PMC fiscal reform. Fig 3 plots the results.

Panels A to C of Fig 3 presents the dynamic impacts of the PMC fiscal reform on newly added firms, zombie firms, and other firms, respectively. The identification method of zombie firms is the Nie method. As shown in Fig 3, coefficients on the PMC fiscal reform dummy variables are insignificantly different from zero for all years before counties adopted the PMC fiscal reform with no trends. Also, newly added firms rise immediately after the implementation of the PMC fiscal reform, zombie firms fall after the implementation of the PMC fiscal reform, and other firms remain unchanged. In sum, the results indicate that changes do not occur before the PMC fiscal reform is adopted, and the impact of the PMC fiscal reform on newly added firms and zombie firms materialized very quickly.

Results in Fig 3 show that local government changed their preference for newly added firms and zombie firms after implementing the PMC fiscal reform, indicating the robustness of our estimation. We get similar results when changing the zombie firm identification method to the Tan method and FN-CHK method (see S1 Appendix). Thus, detecting the mechanisms connecting the PMC fiscal reform and regional development strategy is essential.

### 4.6 Placebo test

In this section, we conduct a placebo test to rule out county-level trends in changes in newly added firms, zombie firms, and other firms and account for the correct structure of the error covariance matrix from the regressions. The placebo test randomizes the assignment of the years that counties adopted the PMC fiscal while keeping the distribution of the event years unchanged [76]. For the randomization, counties that adopted the PMC fiscal reform are randomly selected to get one pseudo county sample and repeat the process 500 times to get 500 pseudo county samples [74]. The baseline regression in each pseudo county sample as columns (1), (2), and (3) in Table 2 have been run, and the relevant coefficients have been saved. Finally, the coefficients from the actual county sample are compared with those from these pseudo county samples.

**Fig 4:**
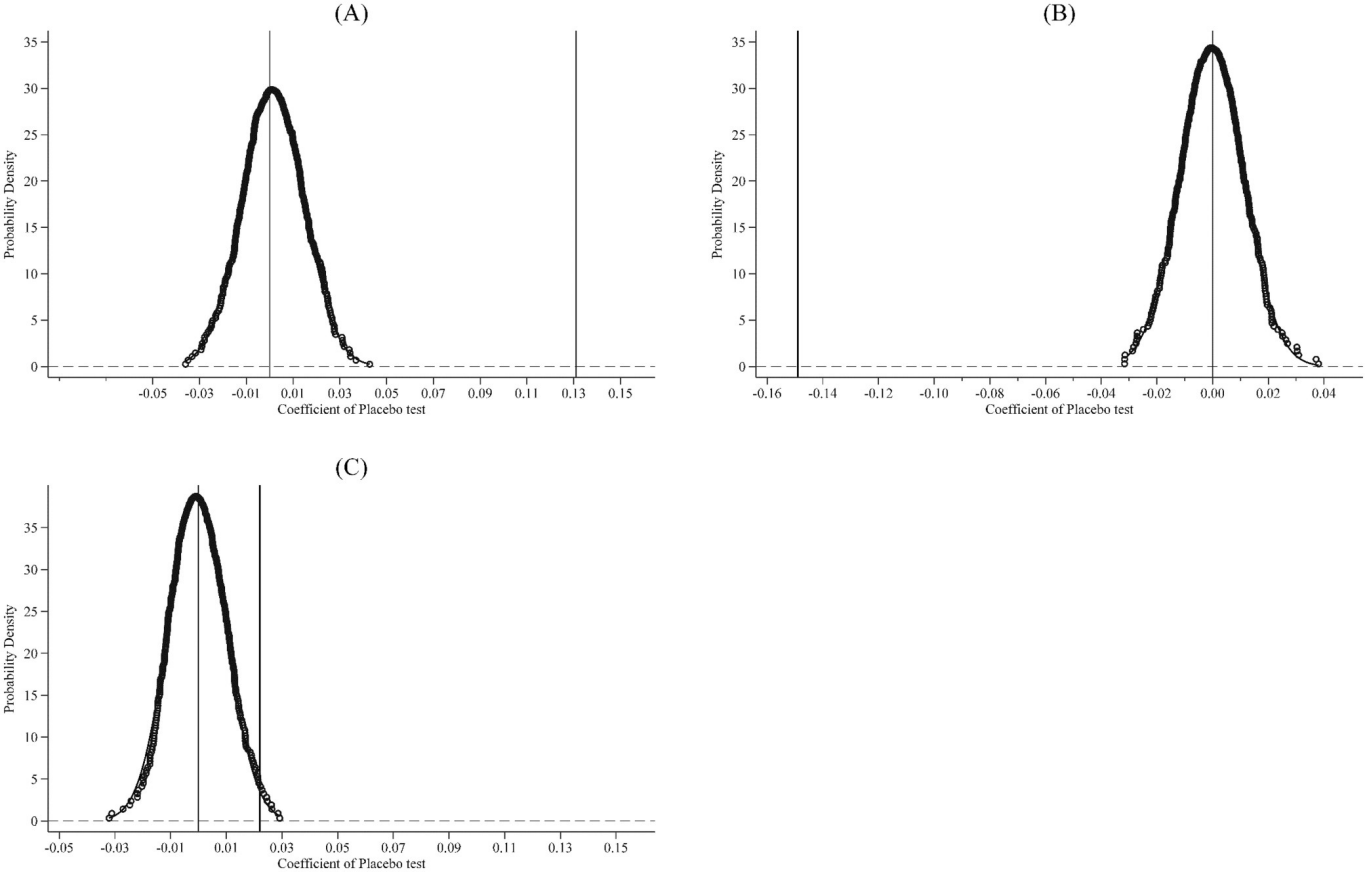
Placebo test of the PMC fiscal reform and regional development strategy. Notes: Panel A is the newly added firms, Panel B is the zombie firms, and Panel C is the other firms. The zombie firm identification method is the Nie method. The figure shows the cumulative distribution density of 500 pseudo estimations. The vertical line presents the result of columns (1), (2), and (3) in Table 2.

The distribution of the 500 pseudo estimations is shown in Fig 4. In Fig 4 Panel A, the black line is the actual estimations, and the graph is the distribution of 500 pseudo estimations. The estimations from random assignments are centered around zero, and the black line, as the benchmark, is located outside the entire distribution suggesting that there is no effect with the randomly constructed county sample. We find the same results in Fig 4 Panel A and Panel B. Also, similar results when changing the zombie firm identification method to the Tan method and FN-CHK method are reported in S1 Appendix. In general, the results of the placebo test suggest that the effects of the PMC fiscal reform on newly added firms, zombie firms, and other firms are not driven by unobserved factors or unmeasured time trends, which gives more assurance regarding the credibility of the results.

## 5. Mechanism

The result presented above shows that the PMC fiscal reform increases newly added firms and decreases zombie firms but has no impact on other firms in the PMC counties. One mechanism that may generate such variation is that the local governments will adjust their strategies of supporting zombie firms and attracting new firms after implementing the PMC fiscal reform. There is a pronounced substitution effect between zombie firms and newly added firms. The PMC fiscal reform may induce local governments’ preference for newly added firms and reduce local governments’ preference for zombie firms, which may reflect in the government subsidy, tax treatment, financial support, et al. This section will not precisely identify how much of the effect on regional development strategy worked through these mechanisms. However, the results build a strong case that the change in the government’s strategy played a critical role.

We also conduct further analysis to understand our study better. We find that the PMC fiscal reform significantly decreases the fiscal burden and improves the fiscal capacity of county-level government. Moreover, the decrease of zombie firms mainly occurs in the state-own enterprises (SOEs), and the increase of newly added firms occurs in the nonstate-owned enterprises (NSOEs). S1 Appendix shows more details about the further analysis, including the impact of PMC fiscal reform on local governments’ fiscal burden and fiscal capacity and the impact of the PMC fiscal reform on zombie firms with different registered types.

### 5.1 Evidence on the government subsidy

The first kind of evidence comes from examining the changes in local government subsidy between newly added firms and zombie firms resulting from the PMC fiscal reform. We use two indicators to measure the substitution of government subsidy, including government subsidy to firms and the number of firms receiving government subsidy with data from CASIF. There is a lack of subsidy data in the CASIF between 2008 and 2010. In this section, we use data from 2000 to 2007.

To examine whether there is a change in the government’s subsidy for newly added firms, zombie firms, and other firms, we estimate similar regressions at the county level by replacing the dependent variables with the government’s subsidy to firms and the number of firms receiving government subsidy plus one, separately. To further prove whether the change of government subsidy preference only occurs in newly added and zombie firms, we also examine the impacts of the PMC fiscal reform on other firms. We also distinguish zombie firms into zombie SOEs and zombie NSOEs. Tables 5 and 6 present the results. The identification method of zombie firms is the Nie method.

**Table 5:** Impacts of the PMC fiscal reform on government subsidy to firms.

	(1)	(2)	(3)	(4)	(5)
**PMC fiscal reform**	-0.605*	-0.674*	0.091	0.433*	0.409
	(0.355)	(0.345)	(0.297)	(0.222)	(0.436)
**Year fixed effect**	Yes	Yes	Yes	Yes	Yes
**County fixed effect**	Yes	Yes	Yes	Yes	Yes
**Control variables**	Yes	Yes	Yes	Yes	Yes
**8 key determinants ×Year**	Yes	Yes	Yes	Yes	Yes
**Treatment trend**	Yes	Yes	Yes	Yes	Yes
**Year coverage**	2000–2007	2000–2007	2000–2007	2000–2007	2000–2007
**Adjusted R-squared**	0.407	0.383	0.281	0.098	0.540
**Obs**.	7563	7563	7563	7563	7563

Notes: In columns (1)-(3), the dependent variables are government subsidy to zombie firms, zombie SOEs and zombie NSOEs, respectively. The identification method of zombie firms is the Nie method. In columns (4)-(5), the dependent variables are government subsidy to newly added firms and other firms, respectively.

*, **, and *** indicate statistical significance at the 10%, 5% and 1% levels respectively.

**Table 6:** Impacts of the PMC fiscal reform on the number of firms receiving government subsidy.

	(1)	(2)	(3)	(4)	(5)
**PMC fiscal reform**	-0.223***	-0.217***	-0.102*	0.253***	0.028
	(0.066)	(0.067)	(0.058)	(0.080)	(0.054)
**Year fixed effect**	Yes	Yes	Yes	Yes	Yes
**County fixed effect**	Yes	Yes	Yes	Yes	Yes
**Control variables**	Yes	Yes	Yes	Yes	Yes
**8 key determinants ×Year**	Yes	Yes	Yes	Yes	Yes
**Treatment trend**	Yes	Yes	Yes	Yes	Yes
**Year coverage**	2000–2007	2000–2007	2000–2007	2000–2007	2000–2007
**Adjusted R-squared**	0.704	0.682	0.668	0.476	0.874
**Obs**.	7563	7563	7563	7369	7563

Notes: In columns (1)-(3), the dependent variables are the number of zombie firms, zombie SOEs and zombie NSOEs receiving government subsidy, respectively. The identification method of zombie firms is the Nie method. In columns (4)-(5), the dependent variables are the number of government subsidy to newly added firms and other firms receiving government subsidy.

*, **, and *** indicate statistical significance at the 10%, 5% and 1% levels respectively.

In Table 5, the significant and negative coefficients in columns (1) and (2) indicate that the total amount of government subsidies to zombie firms and zombie SOEs has decreased, while the insignificant coefficient in column (3) suggests that the total amount of government subsidies to zombie NSOEs have no significant change. In column (4), the significant and positive coefficient indicates that government subsidies to newly added firms have increased. In column (5), the insignificant coefficient shows that government subsidies to other firms have no significant change.

In Table 6, columns (1)-(3), the significant and negative coefficients indicate that the number of zombie firms, zombie SOEs and zombie NSOEs receiving government subsidy has decreased, and the decrease of zombie SOEs is much large than zombie NSOEs. In column (4), the significant and positive coefficient indicates that the number of newly added firms receiving government subsidy has increased, while in column (5), the insignificant coefficient indicates that the number of other firms receiving government subsidy remains unchanged.

Therefore, Tables 5 and 6 show that the PMC fiscal reform reduces the government’s subsidy for zombie firms, especially for zombie SOEs, but increases the subsidy for newly added firms after the PMC fiscal reform. In contrast, the government’s subsidy preference for other firms has no significant change, indicating a shift in the government’s subsidy preference from zombie firms to newly added firms. We change the identification method of zombie firms into the Tan method and the FN-CHK method (see S1 Appendix). The results are nearly the same indicating that the estimation is robust.

### 5.2 Evidence on the tax treatment

Governments often provide favorable tax treatment to SOEs. Lin and Tan [26] show that governments often provide tax reductions to SOEs suffering from losses. Xiao [77] finds that the tax enforcement for NSOEs has increased after the 2002 corporate income tax revenue sharing reform, but not for SOEs. Thus, zombie SOEs usually benefit from these preferential tax treatments from the government. The lower tax burden is one of the important reasons for the survival of zombie SOEs. If the government changes its tax preference for zombie firms, it will not be suitable for the survival of zombie SOEs and will reduce zombie SOEs. Based on this, the second piece of evidence we report focuses on the changes in firms’ tax burden after the PMC fiscal reform. We use data from CASIF of 2000–2013.

We use the ratio of a firm’s corporate income tax to main business income to measure firms’ tax burden. To examine whether there is a change in the tax burden of zombie firms, newly added firms, and other firms, we estimate similar regressions at the county level by replacing the dependent variables with the firm’s corporate income tax to main business income (logarithm). As before, we explore the impacts on zombie firms, zombie SOEs, zombie NSOEs, newly added firms, and other firms to prove whether the change of government preferential tax treatment only occurs in zombie firms. Table 7 presents the results. The identification method of zombie firms is the Nie method.

**Table 7:** Impacts of the PMC fiscal reform on the tax burden of firms.

	(1)	(2)	(3)	(4)	(5)
**PMC fiscal reform**	0.715**	0.974**	0.780**	-0.804***	0.226
	(0.354)	(0.392)	(0.357)	(0.294)	(0.163)
**Year fixed effect**	Yes	Yes	Yes	Yes	Yes
**County fixed effect**	Yes	Yes	Yes	Yes	Yes
**Control variables**	Yes	Yes	Yes	Yes	Yes
**8 key determinants ×Year**	Yes	Yes	Yes	Yes	Yes
**Treatment trend**	Yes	Yes	Yes	Yes	Yes
**Year coverage**	2000–2013	2000–2013	2000–2013	2000–2013	2000–2013
**Adjusted R-squared**	0.316	0.401	0.351	0.215	0.231
**Obs**.	12764	12764	12764	12764	12764

Notes: In columns (1)-(3), the dependent variables are tax burden in zombie firms, zombie SOEs and zombie NSOEs, respectively. The identification method of zombie firms is the Nie method. In columns (4)-(5), the dependent variables are tax burden in newly added firms and other firms, respectively.

*, **, and *** indicate statistical significance at the 10%, 5% and 1% levels respectively.

In Table 7, columns (1) to (3), the significant and positive coefficients indicate that the tax burden on zombie firms, zombie SOEs and zombie NSOEs has increased after the PMC fiscal reform, and the increase of zombie SOEs is much large than zombie NSOEs. In column (4), the significant and negative coefficient indicates that the tax burden on newly added firms has decreased. In column (5), the insignificant coefficients suggest that the tax burden on other firms has no significant change.

Therefore, Table 7 shows that the PMC fiscal reform reduces the government’s tax treatment of zombie firms while increasing the government’s tax treatment of newly added firms, indicating a shift in the government’s tax treatment from zombie firms to newly added firms. This demonstrates that changing the government’s tax treatment of zombie firms and newly added firms is an important mechanism of our main results. We change the identification method of zombie firms into the Tan method and the FN-CHK method (see S1 Appendix). The results are the same indicating that the estimation is robust.

### 5.3 Evidence on the financial support

A study conducted by Faccio [78] shows that when governments decide to rescue particular firms, these firms will enjoy lower interest rates of bank loans. Since the SOEs in China are responsible for the multi-task objectives of governments [79], governments have been willing to rescue troubled SOEs. Also, China’s banking system is dominated by state-owned banks [48]. In these circumstances, zombie SOEs may enjoy lower interest rates of bank loans. Implementing the PMC fiscal reform relieves the fiscal burden of the local governments, thereby dampening governments’ willingness to rescue troubled SOEs. The government’s willingness reduction reflects a higher interest rate of bank loans to SOEs. This, in turn, will reduce the survival of zombie SOEs. These motivate us to test whether the PMC fiscal reform decreases the government’s financial support preference for zombie firms, newly added firms, and other firms.

The third piece of evidence emphasized financial support to zombie and newly added firms using the data from CASIF of 2000–2013. We use the ratio of interest payments to total liabilities to proxy the government’s financial support (logarithm). The identification method of zombie firms is the Nie method. We distinguish firms into zombie firms, zombie SOEs, zombie NSOEs, newly added firms, and other firms. The results of this analysis are presented in Table 8.

**Table 8:** Impacts of the PMC fiscal reform on the government’s financial support to firms.

	(1)	(2)	(3)	(4)	(5)
**PMC fiscal reform**	0.600**	0.928***	0.276	-0.426*	0.058
	(0.262)	(0.304)	(0.266)	(0.244)	(0.103)
**Year fixed effect**	Yes	Yes	Yes	Yes	Yes
**County fixed effect**	Yes	Yes	Yes	Yes	Yes
**Control variables**	Yes	Yes	Yes	Yes	Yes
**8 key determinants ×Year**	Yes	Yes	Yes	Yes	Yes
**Treatment trend**	Yes	Yes	Yes	Yes	Yes
**Year coverage**	2000–2013	2000–2013	2000–2013	2000–2013	2000–2013
**Adjusted R-squared**	0.246	0.303	0.311	0.195	0.197
**Obs**.	12764	12764	12764	12764	12764

Notes: In columns (1)-(3), the dependent variables are the government’s financial support to zombie firms, zombie SOEs and zombie NSOEs, respectively. The identification method of zombie firms is the Nie method. In columns (4)-(5), the dependent variables are the government’s financial support to newly added firms and other firms.

*, **, and *** indicate statistical significance at the 10%, 5% and 1% levels respectively.

In Table 8, columns (1) and (2), the significant positive coefficients of zombie firms and zombie SOEs show that after the PMC fiscal reform, zombie firms, especially zombie SOEs, see their cost of bank loans significantly increase, indicating the decrease in the government’s financial support for zombie SOEs. The insignificant coefficients of zombie NSOEs show that the PMC fiscal reform has no significant effect on the government’s financial support preference for zombie NSOEs. In column (4), the significant negative coefficient of newly added firms shows that the government’s financial support preference for them has increased. In column (5), the insignificant coefficient of other firms shows that the government’s financial support preference has not changed. These results prove that the PMC fiscal reform weakens the government’s financial support for zombie firms, especially zombie SOEs, while strengthening the government’s financial support for newly added firms. We change the identification method of zombie firms into the Tan method and the FN-CHK method (see S1 Appendix). The results are the same indicating that the estimation is robust.

## 6. Conclusion

### 6.1 Discussion of findings

In this paper, we document that fiscal decentralization has notable effects on local governments’ regional development strategy by examining China’s large-scale fiscal decentralization reform, named the Province-Managing-County (PMC) fiscal reform. Our study shows that the PMC fiscal reform has a significantly positive effect on newly added firms, a significantly negative effect on zombie firms, and no effects on other firms. There is a pronounced substitution effect between the strategy of supporting zombie firms and the strategy of attracting new firms. We present evidence supporting this argument, including the substitution effect for subsidies, tax burden, and financial support between newly added firms and zombie firms. Indeed, governments in counties that adopted the PMC fiscal reform may enhance their investment attraction activities and weaken their struggles for zombie firms.

The result of our study is supported by many existing works [12, 80]. Chang et al. [12] find that government intervention, such as subsidies, tax treatment, et al., may trigger zombie firms, which proves the conclusion of our study from the opposite side. He and Sun [80] show that fiscal decentralization promotes investment inflow. Existing studies have either conducted research from the perspective of zombie firms or undertaken only research from the perspective of attracting investment and new firms. Our study is different from the existing works by considering the government’s choice of zombie firms and new firms in the same analysis framework, which studies the subject of relationships between government and firms.

### 6.2 Recommendation

These conclusions have some implications. First, policymakers must be aware that intergovernmental fiscal relations would affect local governments’ regional development strategies. Intergovernmental fiscal relations are closely linked with local governments’ fiscal pressure and capacity, which may motivate them to adjust their regional development strategy.

Second, increasing the fiscal autonomy of local governments can be an effective way to motivate the local government to start a more sustainable and healthier economy. When local governments lack fiscal autonomy, they often keep zombie firms afloat to maintain economic stability. In fact, local governments in China can play the role of development-oriented governments [81], but their fiscal autonomy needs to be enhanced. Our study shows that the PMC fiscal reform-induced increases in fiscal autonomy have reduced zombie firms and increased new firms. The increase of new firms and the disposition of zombie firms can reduce resource misallocation, thus making economic development healthier and more sustainable.

Last, the central government should provide necessary support such as fiscal transfers or political incentives to stimulate the local governments to optimize their regional development strategy. Although local governments must make appropriate regional development strategies based on local conditions and pursue sustainable development, incentives from the central government are also needed.

## Supporting information

S1 Appendix (DOCX)
