# Supplementary material for: Like the new and hate the old: The impact of fiscal decentralization on regional development strategy
Source: PLoS One. 2022 Sep 9;17(9):e0273875. doi: 10.1371/journal.pone.0273875 (PMC9462755; doi:10.1371/journal.pone.0273875)
Supplement: S1 Appendix — (DOCX) [file pone.0273875.s001.docx]

### The PMC fiscal reform in China

Fiscal decentralization refers to transferring fiscal power and responsibility from central to sub-national governments. Although China is a unitary state, China’s fiscal system is generally characterized by fiscal decentralization. Sub-national governments in China manage local fiscal affairs on behalf of the central government. There are four layers of China’s sub-national governments, from the provincial-level government, through the prefectural-level government and county-level government, to the township-level government. Each subnational level of government is subordinated to the next higher order of government. In practice, the provincial-level government owns the fiscal authority of the prefectural-level government, and by that analogy, the prefectural-level government owns the fiscal authority of the county-level government. There is no direct linkage between the provincial-level government and the county-level government. This approach is termed the prefecture-managing-county fiscal system.

However, with the rapid growth of the Chinese economy, drawbacks of the prefecture-managing-county fiscal system appear gradually. The prefecture-managing-county fiscal system hampered administrative efficiency and resulted in a widening gap between revenue and expenditure assignments at the county level [1]. On the one hand, the prefecture-managing-county fiscal system grants prefecture-level governments the authority on county affairs. County-level governments may lose their independence to devise appropriate policies for their jurisdictional development and implement them based on local conditions [2]. Also, concerning their interests, prefecture-level governments are incentivized to distort top-down communication or bottom-up reporting between provincial and county governments. This system makes the county-level governments lack the authority of county fiscal affairs and reduces administrative efficiency.

On the other hand, since prefectural-level governments own the discretion to set up their intergovernmental fiscal relationships within counties [2], they may tend to centralize fiscal funds at the prefectural level at the expense of the counties. Meanwhile, county-level governments have been responsible for regional social and economic development expenditures and public services. Therefore, county-level governments usually suffer from gaps between revenue and expenditure and face fiscal distress. After implementing the tax-sharing fiscal system (TSS) in 1994, which strengthened the central government’s control over tax revenue, the fiscal distress of county-level governments became more and more outstanding.

As a result, China has initiated the “Province-Managing-County” (PMC) fiscal reform as a strategy to overcome the challenges resulting from the prefecture-managing-county fiscal system. It is first implemented in Zhejiang province and Hainan province as early as the 1980s. It has been gradually implemented in other provinces across China since 2004. However, unlike Zhejiang and Hainan provinces, where all counties have adopted the PMC fiscal reform, other provinces selected some counties to adopt the PMC fiscal reform. The central-level government specified that counties with large grain, oil plants, and cotton own the priority in implementing the PMC fiscal reform [2]. Besides the essential guidance, province-level governments also treat economic scale, poverty, development potential, and criteria to select counties for PMC fiscal reform. By the end of 2012, 24 provinces in China have implemented the PMC fiscal reform [3].

The development of the PMC fiscal reform can be divided into three stages. The first stage is the exploration stage from the 1980s to 2003, which is viewed as a preliminary exploration of fiscal decentralization reform. Only Zhejiang and Hainan have carried out the PMC fiscal reform during the period (Actually, based on Li et al. [4], we exclude Hainan and Zhejiang from the sample to keep the robustness of the results).

The second stage is the pilot stage from 2004 to 2013. Since 2004, Hubei Province, Henan Province, and other provinces in China began to adopt the PMC fiscal reform gradually. Then, the national 11th Five-Year Plan, released in 2006, pointed out that the reform of government institutions should be deepened and the administrative level should be lowered, creating favorable policy conditions for the PMC fiscal reform. In 2009, the Ministry of Finance issued “Opinions on Boosting the Province-Managing-County Fiscal Reform”, which proposed that, except for ethnic autonomous regions, the whole country should strive to comprehensively promote the PMC fiscal reform by the end of 2012. Thus, before 2013, the PMC fiscal reform had been piloted nationwide.

The third stage is the adjustment stage from 2014 to now. Since 2014, governments have begun to rethink the PMC fiscal reform. Local governments’ opinions have polarized the PMC fiscal reform. For example, Hunan and Shanxi provinces still promote the PMC fiscal reform and expand the scope of the reform, while Hebei provinces have suspended the reform in some counties.

According to the development process of reform, at present, the PMC fiscal reform is in a period of adjustment. Therefore, it is very important to learn from the experience of previous reform for how to optimize reform at the adjustment stage. The study period of our study is almost in the pilot stage of the PMC fiscal reform. Therefore, our research results can provide enlightenment for the PMC fiscal reform at the adjustment stage.

The PMC fiscal reform mainly contains two aspects. First, the PMC fiscal reform removes the existing fiscal relationships between prefectural and county-level governments. It places the county-level government’s fiscal authority under the exclusive control of the provincial-level government. The PMC fiscal reform established a direct link between the provincial-level governments and the county-level governments. In practice, fiscal affairs in counties that adopted the PMC fiscal reform are directly managed by the provincial-level government, including but not limited to revenue and expenditure assignments, intergovernmental transfers, government budget, and final accounts [3]. While the counties have not adopted the PMC reform, the prefectural-level government still manages its fiscal affairs.

Second, the PMC fiscal reform adjusts the assignments of revenues and spending and the relationship on intergovernmental fiscal transfer between the county-level government and the prefecture-level government, which restores the power of county-level governments to make independent fiscal decisions and eases the fiscal burden of county-level governments (Table.S1 lists the related documents of the PMC fiscal reform). Although measures of the PMC fiscal reform may vary from place to place, the core measures are consistent. The three main points are these:

(1) The assignments of revenues between the county-level government and the prefecture-level government are adjusted. Indeed, the fiscal resources available to county-level governments are often limited before the PMC fiscal reform. There are two main reasons. In the prescribed proportion, the county-level government must turn over revenues to the central government, the provincial-level government, and the prefecture-level government. Second, the prefecture-level government will likely retain revenues that should have belonged to the county-level governments in further development. The PMC fiscal reform assigns revenues between the prefecture-level government and the county-level government in a more reasonable way, which may increase the revenue of the county-level government, and decrease its fiscal pressure.

(2) The spending responsibilities between the county and prefecture governments have been standardized and adjusted. To relieve fiscal pressure, the prefecture-level government may shift its spending responsibilities to the subordinate county-level government. The PMC fiscal reform solves this problem by assigning spending responsibilities to the county and prefecture governments. To some extent, the PMC fiscal reform reduces the spending burden of the county-level government and relieves their fiscal pressure.

(3) The intergovernmental fiscal transfers and tax rebates to the county-level governments are no longer transferred from the prefecture-level governments to the county-level governments but directly from the provincial-level government to the county-level government. So, the prefecture-level governments can no longer retain fiscal transfers and tax rebates that belong to county-level governments. Studies show that the PMC fiscal reform significantly increases the fiscal transfers received by PMC counties [2], thus improving county-level governments’ fiscal capacities.

Table. The related documents of the PMC fiscal reform

| **Province** | **Official document** | **Key points on fiscal decentralization** |
| --- | --- | --- |
| Gansu province | Notice of the People’s Government of Gansu Province on Issuing the Pilot Plan for implementing the Province-Managing-County Fiscal Reform in Gansu Province | 1. Revenue and expenditure:  a) In the pilot PMC counties, the income sharing methods on the administrative fee, special income, and other income between the county and prefecture-level governments are canceled. Administrative fees, special income, and other income shall be subdivided into the central governments, provincial-level governments, and county-level governments according to relevant central and provincial regulations.  b) In the pilot PMC counties, the income sharing methods on revenues from government funds are canceled. The revenues from government funds shall be subdivided into the central governments, provincial-level governments, and county-level governments according to relevant central and provincial regulations.  2. Intergovernmental fiscal transfer: Intergovernmental fiscal transfers in the pilot PMC counties are directly distributed to county-level governments. |
| Henan Province | Notice of the People’s Government of Henan Province on Improving the Fiscal System between Provinces and Cities and Counties | Intergovernmental fiscal transfer:  a) Increase the proportion coefficient of the cities’ and counties’ “two taxes” refund growth (two taxes referring to consumption tax and value-added tax).  b) According to the calculation of the province’s general intergovernmental fiscal transfers to cities and counties in 2008, for cities and counties with basic expenditure gaps, all the gaps are included in the county and city subsidy base. |
| Jilin province | Notice of the People’s Government of Jilin Province on Implementing the Province-Managing-County Fiscal Reform in Gansu Province | 1. Revenue and expenditure: The division of powers or authority of office between governments at all levels determines the spending responsibilities between the provincial, prefecture, and county governments.  2. Intergovernmental fiscal transfer: Governmental fiscal transfers from the provincial-level government to the prefecture-level government and county-level government are directly delegated to the prefecture-level government and county-level government after implementing the PMC fiscal reform. |
| Liaoning province | Notice of the General Office of the People’s Government of Liaoning Province on the Implementation of the Province-Managing-County Fiscal Reform for Suizhong County | 1. Revenue and expenditure:  a) Huludao City will no longer share the revenues of Suizhong County.  b) The collection and management authority of various non-tax and government fund revenues are decentralized from the prefecture-level government to the county-level government. Except for non-tax and government fund revenues that need to be turned over to the central government and provincial-level government as required by regulations, the rest are treated as county-level government revenues. The prefecture-level government no longer participates in the sharing.  2. Intergovernmental fiscal transfer: The Suizhong County government applies to the provincial-level government for intergovernmental special fiscal transfers, and the provincial-level government directly issues to Suizhong County. |
| Shandong Province | Notice of the People’s Government of Shandong Province on Implementing the Pilot Province-Managing-County Fiscal Reform | 1. Revenue and expenditure: By the territorial division of income, the scope and proportion of income sharing at the central government and provincial-level government under the current system will remain unchanged. Prefecture-level cities with districts will no longer participate in sharing tax revenue and various non-tax revenues of the PMC counties.  2. Intergovernmental fiscal transfer: Intergovernmental fiscal transfer from the provincial-level government to the county-level government shall be individually approved to reach the PMC counties. |
| Shanxi Province | Notice of the People’s Government of Shanxi Province on Adjusting and Regulating the Financial System of Provinces, Cities, and Counties and Implementing the Pilot Province-Managing-County Fiscal Reform in 35 Key National Poverty Alleviation and Development Counties | 1. Revenue and expenditure: The scope of tax revenue sharing in provinces, prefecture-level cities, and counties in the local retained part of five tax types: value-added tax, business tax, resource tax, corporate income tax, and personal income tax (from now on referred to as the “five taxes”). All urban land use taxes are decentralized as a prefecture-level city and county revenue. The five tax sharing ratios are 30% at the provincial-level government, 15% at the prefecture-level government, and no less than 55% at the county-level government.  2. Intergovernmental fiscal transfer: Establish a provincial-to-prefecture general fiscal transfer system to ensure the financial needs for the operation of the prefecture-level government and career development. |
| Shaanxi Province | Notice of the People’s Government of Shaanxi Province on Implementing the Pilot Province-Managing-County Fiscal Reform | 1. Revenue and expenditure: The provincial and county fiscal revenue scope will not be adjusted. The prefecture-level cities will no longer participate in the county’s tax revenue sharing and will no longer concentrate the county’s new financial resources.  2. Intergovernmental fiscal transfer: All the intergovernmental fiscal transfers are directly subsidized to the county. |
| Yunnan Province | Notice of the People’s Government of Yunnan Province on Implementing the Pilot Province-Managing-County Fiscal Reform | 1. Revenue and expenditure: By the relevant regulations of the tax-sharing fiscal system and the principle of territoriality, fiscal revenues realized within the pilot PMC counties will be shared by the central government, provincial-level government, and county-level government. All revenue-sharing policies of the prefecture-level city to the pilot PMC counties shall be suspended.  2. Intergovernmental fiscal transfer: Intergovernmental fiscal transfers from the provincial-level government are subsidized to the pilot PMC counties. |
| Hunan Province | Notice of the Hunan Provincial Committee of the Communist Party of China and the Hunan Provincial People’s Government on Improving the Financial System and Implementing the Province-Managing-County Fiscal Reform | 1. Revenue and expenditure:  a) The local part of value-added tax and business tax are included in the scope of sharing and will be shared between provinces and prefecture-level cities or between provinces and counties (county-level cities) on a proportional basis. The local part of corporate income tax and the local part of personal income tax is adjusted from sharing between provinces and prefecture-level cities to sharing between provinces and prefecture-level cities or provinces and counties (county-level cities). Adjust the sharing ratio of resource tax.  b) After the PMC reform, prefecture-level cities and counties (county-level cities) became independent of the financial management system. Prefecture-level cities with districts will no longer share the revenues of their affiliated counties and county-level cities.  2. Intergovernmental fiscal transfer:  a) Intergovernmental fiscal transfers from the provincial-level government are directly subsidized to prefecture-level cities and counties (county-level cities).  b) Adjust the province’s “two taxes” (value-added tax and consumption tax) refunds from the provincial-level government to prefecture-level cities and counties (county-level cities). |
| Anhui Province | Notice of the People’s Government of Anhui Province on Implementing the Province-Managing-County Fiscal Reform | 1. Revenue and expenditure: By the provisions of the fiscal system further strictly regulate the scope of fiscal revenues and expenditure of the prefecture-level and county-level governments.  2. Intergovernmental fiscal transfer: Intergovernmental fiscal transfers from provincial-level government to county-level government are uniformly calculated and subsidized to counties. |
| Guangxi Province | Notice of the People’s Government of Guangxi Zhuang Autonomous Region on Implementing the Pilot Province-Managing-County Fiscal Reform | 1. Revenue and expenditure: Provincial-level government shall standardize and appropriately adjust the division of revenues and expenditure between prefecture-level government and county-level government that does not meet the requirements for supporting county economic development.  2. Intergovernmental fiscal transfer: Intergovernmental fiscal transfer from provincial-level government to county-level government and tax rebates will be directly approved by the provincial-level government and subsidized to pilot PMC counties. |
| Jiangsu Province | Notice of the People’s Government of Jiangsu Province on Implementing the Province-Managing-County Fiscal Reform | 1. Revenue and expenditure:  a) The provincial-level government shall adjust the division of revenue and expenditure between prefecture-level government and county-level government, which does not meet the requirements.  b) After implementing the PMC fiscal reform, the prefecture-level government shall not assign income that should fall within the scope of county-level government to the prefecture-level government and shall not centralize county-level government fiscal revenue and funds in any way.  c) After implementing the PMC fiscal reform, the prefecture-level government shall not transfer the expenditure responsibilities to the county-level government that the prefecture-level government should undertake.  2. Intergovernmental fiscal transfer: Improve the incentive mechanism for accelerating county development and promoting the mutual development of prefecture-level cities and counties. Increase transfer payments, consider areas converted from counties with weak economies into districts, and ease the financial difficulties of counties and townships. |
| Qinghai Province | The General Office of the People’s Government of Qinghai Province forwarded the notice of the Finance Department of Qinghai Province on the Opinions on the pilot Province-Managing-County Fiscal Reform | Intergovernmental fiscal transfer:  a) Intergovernmental fiscal transfer from provincial-level government to the county-level government will be directly approved by the provincial-level government and subsidized to pilot PMC counties.  b) To ensure that the vested financial interests of the prefecture-level government are not affected, the provincial finance will subsidize the prefecture-level government by general intergovernmental fiscal transfer according to the regulations and the financial difficulties at the prefecture-level government.  c) To prevent the prefecture-level government from centralizing county-level government financial resources through system adjustment, the province-level government will reward and punish the prefecture-level government through incentive intergovernmental fiscal transfer. |
| Hebei Province | Notice of the People’s Government of Hebei Province on Implementing the Province-Managing-County Fiscal Reform | Revenue and expenditure:  a) After the PMC fiscal reform, prefecture-level cities with districts will no longer participate in the sharing of the revenues of the PMC counties in principle.  b) By the unification of responsibilities and powers principle, prefecture-level governments with districts will no longer bear the corresponding additional expenditure responsibilities of the PMC counties. |
| Heilongjiang Province | Implementation Opinions of the People’s Government of Heilongjiang Province on Strengthening Financial Support to Accelerate the Development of County Economy | 1. Revenue and expenditure: The increased fiscal revenue of each county shall be entirely at the county’s disposal, and the county itself shall absorb the additional expenditure except for policy-induced expenditure.  2. Intergovernmental fiscal transfer: Increase the proportion of tax rebates. |
| Hubei Province | Opinions on Further Improving the Province-Managing-County Fiscal System and Counties and Strengthening Financial Management | Intergovernmental fiscal transfer: To mobilize the enthusiasm of the prefecture-level governments in supporting the development-affiliated counties, the incentive intergovernmental fiscal transfer policy is implemented by the prefecture-level governments who support the development-affiliated counties. |

### Zombie firm identification methods

In particular, Caballero et al. [5] define the hypothetical risk-free interest payments as follows:

$I_{i,t}^{*}=r_{t-1}^{S}B_{i, t-1}^{S}+\left( \frac{1}{5}\sum_{j=1}^{5} r_{t-j}^{L} \right)B_{i, t-1}^{L}+{rcb}_{\min over last 5 years,t}\times{Bonds}_{i,t-1}$ (1)

where $I_{i,t}^{*}$ is the hypothetical risk-free interest payments. $B_{i, t}^{S}$, $B_{i, t}^{L}$, and ${Bonds}_{i,t}$ are short-term borrowings from banks, long-term borrowings from banks, and total bonds outstanding of firm *i* at the end of year *t*. $r_{t}^{S}$ and $r_{t}^{L}$ are the average short-term and long-term prime rates for year t, respectively. ${rcb}_{\min over last 5 years,t}$ is the minimum rate on a convertible corporate bond issued over the previous five years before year t. Shot-term means less than one year, and long-term means over one year. The drawback of this approach is that it may produce misjudgment or omission of zombie firms.

### Dynamics of the PMC fiscal reform and zombie firm by using the Tan and FN-CHK methods


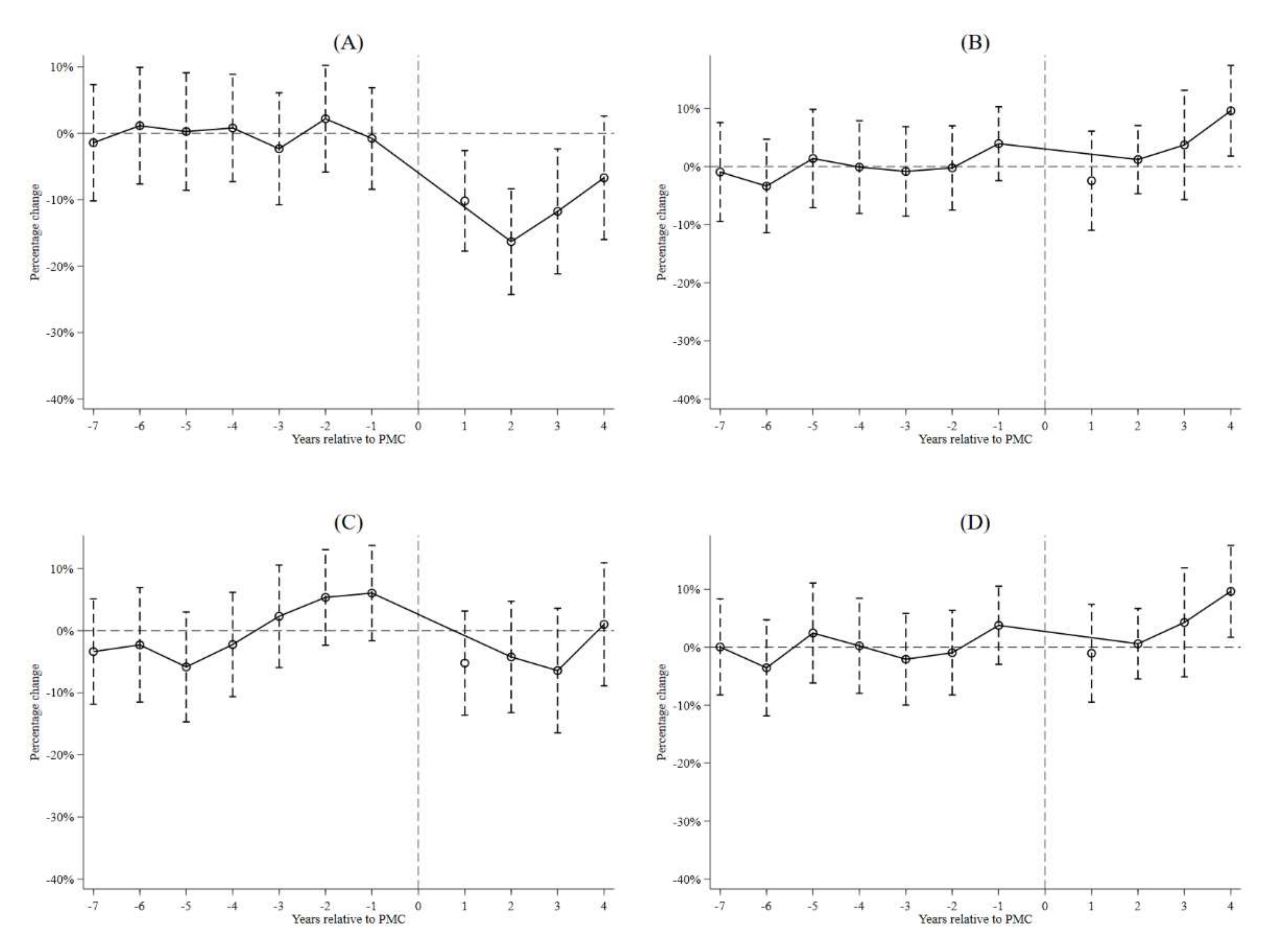


Notes: The figure plots the impact of the PMC fiscal reform on zombie firms. Panel A and Panel B are the zombie firms and the other firms by using the Tan method, Panel C and Panel D are the zombie firms, and the other firms by using the FN-CHK method. All observations are at the county–year level. The dashed lines represent 95% confidence intervals.

### Placebo test of the PMC fiscal reform and the zombie firms using the Tan and FN-CHK methods





Notes: The figure plots the results of the placebo test. The dependent variables are the natural log of zombie firms. Panel A and Panel B are the zombie firms and the other firms by using the Tan method, Panel C and Panel D are the zombie firms, and the other firms by using the FN-CHK method. All observations are at the county–year level. The figure shows the cumulative distribution density of 500 pseudo estimations. The vertical line presents the result of columns (4) to (7) in Table 2, respectively.

### Further analysis: How the PMC fiscal reform affects local governments’ fiscal burden and fiscal capacity

We first explore the impacts of the PMC fiscal reform on county-level governments’ fiscal burden. We use three different indicators as proxy variables of county-level governments’ fiscal burden, including the ratio of local fiscal expenditure to revenue (referring to Huang & Du [6]), the ratio of the gap between local government expenditure and revenue to the revenue (referring to [2]), and the ratio of the gap between local government expenditure and revenue to the GDP (referring to [7] and [8]). Columns (1)-(3) of Table S2 report the impact of the PMC fiscal reform on the fiscal burden of local governments. The results show that the PMC fiscal reform has a significant negative impact on the fiscal burden of county-level governments, indicating that the PMC fiscal reform significantly reduces the fiscal burden of county-level governments.

Then we explore the impacts of the PMC fiscal reform on county-level governments’ fiscal capacity. We use four different indicators as proxy variables of county-level governments’ fiscal capacity, including the fiscal self-sufficiency rate measured by the ratio of local fiscal revenue to expenditure, the ratio of local fiscal revenue to GDP, the local fiscal revenue per capita, and the local intergovernmental fiscal transfer per capita (including the intergovernmental fiscal transfer payments and the tax rebate). Columns (4)-(7) of Table S2 report the impact of PMC fiscal reform on the fiscal capacity of local governments. The significant positive results show that the PMC fiscal reform has significantly improved the fiscal capacity of county-level government. The results in Table S2 show that the PMC fiscal reform significantly decreases the fiscal burden and improves the fiscal capacity of county-level government.

Table S2. How the PMC fiscal reform affects local governments’ fiscal burden and fiscal capacity

|  | **(1)** | **(2)** | **(3)** | **(4)** | **(5)** | **(6)** | **(7)** |
| --- | --- | --- | --- | --- | --- | --- | --- |
| **PMC fiscal reform** | -1.083** | -1.083** | -0.020* | 0.072** | 0.086*** | 0.078*** | 0.024* |
|  | (0.439) | (0.439) | (0.012) | (0.030) | (0.025) | (0.025) | (0.014) |
| **Year fixed effect** | Yes | Yes | Yes | Yes | Yes | Yes | Yes |
| **County fixed effect** | Yes | Yes | Yes | Yes | Yes | Yes | Yes |
| **Control variables** | Yes | Yes | Yes | Yes | Yes | Yes | Yes |
| **8 key determinants ×Year** | Yes | Yes | Yes | Yes | Yes | Yes | Yes |
| **Treatment trend** | Yes | Yes | Yes | Yes | Yes | Yes | Yes |
| **Year coverage** | 2000-2013 | 2000-2013 | 2000-2013 | 2000-2013 | 2000-2013 | 2000-2013 | 2000-2009 |
| **Adjusted R-squared** | 0.754 | 0.754 | 0.827 | 0.854 | 0.744 | 0.914 | 0.944 |
| **Obs.** | 12676 | 12676 | 12676 | 12676 | 12680 | 12680 | 9389 |

Notes: Columns (1)-(3) report the impact of PMC fiscal reform on the ratio of local fiscal expenditure to revenue, the ratio of the gap between local government expenditure and revenue to the revenue, and the ratio of the gap between local government expenditure and revenue to the GDP. Columns (4)-(7) report the impact of PMC fiscal reform on the ratio of local fiscal revenue to expenditure, the ratio of local fiscal revenue to GDP, the local fiscal revenue per capita, and the local intergovernmental fiscal transfer per capita. *, **, and *** indicate statistical significance at the 10%, 5% and 1% levels respectively.

### Further analysis: Impact of the PMC fiscal reform on zombie firms with different registered types

The number of zombie firms in China varies in firms with different registered types. For instance, Shen and Chen [9] study the distribution of zombie firms across firms with different registered types using China Annual Surveys of Industrial Firms (CASIF) and show that, in SOEs, the proportion of zombie firms is 11%, while in NSOEs, the proportion of zombie firms is less than 6%. So we want to determine whether the impacts of the PMC fiscal reform will also vary in firms with different registered types. To clarify, we next divide newly added and zombie firms into two major categories according to different firms’ registered types [10], including SOEs and NSOEs.

Table S3 reports the results of the PMC fiscal reforms on newly added SOEs in column (1), newly added NSOEs in column (2), zombie SOEs in columns (3), (5), and (7), and zombie NSOEs in columns (4), (6) and (8). The methods used to identify zombie firms in columns (3)-(4) are the Nie method, columns (5)-(6) are the Tan method, and columns (7)-(8) are the FN-CHK method. Results in columns (1)-(2) show that the PMC fiscal reform has no impact on newly added SOEs but a significant positive impact on newly added NSOEs. Results in columns (3)-(6) show that the PMC fiscal reform has a significant negative impact on zombie SOEs and zombie NSOEs, while the impact on zombie SOEs is much larger than on zombie NSOEs. Results in columns (7)-(8) show that the PMC fiscal reform has a significant negative impact on zombie SOEs while having little impact on zombie NSOEs. In general, results in Table S3 indicate that the increase of newly added NSOEs mainly drives the positive impact of PMC fiscal reform on newly added firms, and the negative impact of PMC fiscal reform on zombie firms is mainly driven by the reduction of zombie SOEs.

Table S3. The impact of the PMC fiscal reform on firms with different registered types.

|  | **(1)** | **(2)** | **(3)** | **(4)** | **(5)** | **(6)** | **(7)** | **(8)** |
| --- | --- | --- | --- | --- | --- | --- | --- | --- |
| **PMC fiscal reform** | 0.013 | 0.075* | -0.123*** | -0.083* | -0.129*** | -0.079* | -0.073** | -0.072 |
|  | (0.014) | (0.039) | (0.039) | (0.044) | (0.039) | (0.045) | (0.036) | (0.050) |
| **Year fixed effect** | Yes | Yes | Yes | Yes | Yes | Yes | Yes | Yes |
| **County fixed effect** | Yes | Yes | Yes | Yes | Yes | Yes | Yes | Yes |
| **Control variables** | Yes | Yes | Yes | Yes | Yes | Yes | Yes | Yes |
| **8 key determinants ×Year** | Yes | Yes | Yes | Yes | Yes | Yes | Yes | Yes |
| **Treatment trend** | Yes | Yes | Yes | Yes | Yes | Yes | Yes | Yes |
| **Year coverage** | 2000-2013 | 2000-2013 | 2000-2013 | 2000-2013 | 2000-2013 | 2000-2013 | 2000-2013 | 2000-2013 |
| **Adjusted R-squared** | 0.084 | 0.810 | 0.682 | 0.629 | 0.679 | 0.626 | 0.716 | 0.702 |
| **Obs.** | 12764 | 12764 | 12764 | 12764 | 12764 | 12764 | 12764 | 12764 |

Notes: Columns (1)-(2) report the effects of the PMC fiscal reform on newly added SOEs and newly added NSOEs, respectively. The zombie firm identification method in columns (3)-(4) is the Nie method, which reports the effects of the PMC fiscal reform on zombie SOEs and zombie NSOEs, respectively. The zombie firm identification method in columns (5)-(6) is the Tan method, which reports the effects of the PMC fiscal reform on zombie SOEs and zombie NSOEs, respectively. The zombie firm identification method in columns (7)-(8) is the FN-CHK method, which reports the effects of the PMC fiscal reform on zombie SOEs and zombie NSOEs, respectively. *, **, and *** indicate statistical significance at the 10%, 5% and 1% levels respectively.

### Impacts of the PMC fiscal reform on government subsidy to firms by using the Tan Method

|  | **(1)** | **(2)** | **(3)** | **(4)** | **(5)** |
| --- | --- | --- | --- | --- | --- |
| **PMC fiscal reform** | -0.640* | -0.693* | -0.084 | 0.433* | 0.726* |
|  | (0.368) | (0.370) | (0.306) | (0.222) | (0.423) |
| **Year fixed effect** | Yes | Yes | Yes | Yes | Yes |
| **County fixed effect** | Yes | Yes | Yes | Yes | Yes |
| **Control variables** | Yes | Yes | Yes | Yes | Yes |
| **8 key determinants ×Year** | Yes | Yes | Yes | Yes | Yes |
| **Treatment trend** | Yes | Yes | Yes | Yes | Yes |
| **Year coverage** | 2000-2007 | 2000-2007 | 2000-2007 | 2000-2007 | 2000-2007 |
| **Adjusted R-squared** | 0.439 | 0.405 | 0.329 | 0.098 | 0.527 |
| **Obs.** | 7563 | 7563 | 7563 | 7563 | 7563 |

Notes: The table presents the estimates of the impact of the PMC fiscal reform on government subsidy to firms using the Tan method. In columns (1)-(3), the dependent variables are government subsidy to zombie firms, zombie SOEs, and zombie NSOEs, respectively. The identification method of zombie firms is the Nie method. In columns (4)-(5), the dependent variables are government subsidy to newly added firms and other firms (firms excluding zombie firms and newly added firms), respectively. All observations are at the county-year level. *, **, and *** indicate statistical significance at the 10%, 5% and 1% levels respectively.

### Impacts of the PMC fiscal reform on government subsidy to firms by using the FN-CHK method

|  | **(1)** | **(2)** | **(3)** | **(4)** | **(5)** |
| --- | --- | --- | --- | --- | --- |
| **PMC fiscal reform** | -0.761* | -0.815** | 0.032 | 0.433* | 0.859** |
|  | (0.390) | (0.355) | (0.354) | (0.222) | (0.420) |
| **Year fixed effect** | Yes | Yes | Yes | Yes | Yes |
| **County fixed effect** | Yes | Yes | Yes | Yes | Yes |
| **Control variables** | Yes | Yes | Yes | Yes | Yes |
| **8 key determinants ×Year** | Yes | Yes | Yes | Yes | Yes |
| **Treatment trend** | Yes | Yes | Yes | Yes | Yes |
| **Year coverage** | 2000-2007 | 2000-2007 | 2000-2007 | 2000-2007 | 2000-2007 |
| **Adjusted R-squared** | 0.443 | 0.399 | 0.354 | 0.098 | 0.530 |
| **Obs.** | 7563 | 7563 | 7563 | 7563 | 7563 |

Notes: The table presents the estimates of the impact of the PMC fiscal reform on government subsidy to firms. In columns (1)-(3), the dependent variables are government subsidy to zombie firms, zombie SOEs, and zombie NSOEs, respectively. The identification method of zombie firms is the Nie method. In columns (4)-(5), the dependent variables are government subsidy to newly added firms and other firms (firms excluding zombie firms and newly added firms), respectively. All observations are at the county-year level. *, **, and *** indicate statistical significance at the 10%, 5% and 1% levels respectively.

### Impacts of the PMC fiscal reform on the number of firms receiving government subsidy by using the Tan method.

|  | **(1)** | **(2)** | **(3)** | **(4)** | **(5)** |
| --- | --- | --- | --- | --- | --- |
| **PMC fiscal reform** | -0.253*** | -0.252*** | -0.113* | 0.253*** | 0.033 |
|  | (0.066) | (0.068) | (0.060) | (0.080) | (0.053) |
| **Year fixed effect** | Yes | Yes | Yes | Yes | Yes |
| **County fixed effect** | Yes | Yes | Yes | Yes | Yes |
| **Control variables** | Yes | Yes | Yes | Yes | Yes |
| **8 key determinants ×Year** | Yes | Yes | Yes | Yes | Yes |
| **Treatment trend** | Yes | Yes | Yes | Yes | Yes |
| **Year coverage** | 2000-2007 | 2000-2007 | 2000-2007 | 2000-2007 | 2000-2007 |
| **Adjusted R-squared** | 0.707 | 0.683 | 0.668 | 0.476 | 0.874 |
| **Obs.** | 7563 | 7563 | 7563 | 7369 | 7563 |

Notes: The table presents the estimates of the impact of the PMC fiscal reform on the number of firms receiving government subsidy. In columns (1)-(3), the dependent variables are the number of zombie firms, zombie SOEs, and zombie NSOEs receiving government subsidy. The identification method of zombie firms is the Nie method. In columns (4)-(5), the dependent variables are the number of government subsidy to newly added firms and other firms (firms excluding zombie firms and newly added firms) receiving government subsidy, respectively. All observations are at the county-year level. *, **, and *** indicate statistical significance at the 10%, 5% and 1% levels respectively.

### Impacts of the PMC fiscal reform on the number of firms receiving government subsidy by using the FN-CHK method

|  | **(1)** | **(2)** | **(3)** | **(4)** | **(5)** |
| --- | --- | --- | --- | --- | --- |
| **PMC fiscal reform** | -0.158** | -0.170** | -0.074 | 0.253*** | 0.051 |
|  | (0.066) | (0.067) | (0.070) | (0.080) | (0.055) |
| **Year fixed effect** | Yes | Yes | Yes | Yes | Yes |
| **County fixed effect** | Yes | Yes | Yes | Yes | Yes |
| **Control variables** | Yes | Yes | Yes | Yes | Yes |
| **8 key determinants ×Year** | Yes | Yes | Yes | Yes | Yes |
| **Treatment trend** | Yes | Yes | Yes | Yes | Yes |
| **Year coverage** | 2000-2007 | 2000-2007 | 2000-2007 | 2000-2007 | 2000-2007 |
| **Adjusted R-squared** | 0.726 | 0.707 | 0.730 | 0.476 | 0.873 |
| **Obs.** | 7563 | 7563 | 7563 | 7369 | 7563 |

Notes: The table presents the estimates of the impact of the PMC fiscal reform on the number of firms receiving government subsidy. In columns (1)-(3), the dependent variables are the number of zombie firms, zombie SOEs, and zombie NSOEs receiving government subsidy. The identification method of zombie firms is the Nie method. In columns (4)-(5), the dependent variables are the number of government subsidy to newly added firms and other firms (firms excluding zombie firms and newly added firms) receiving government subsidy, respectively. All observations are at the county-year level. *, **, and *** indicate statistical significance at the 10%, 5% and 1% levels respectively.

### Impacts of the PMC fiscal reform on the tax burden of firms by using the Tan method

|  | **(1)** | **(2)** | **(3)** | **(4)** | **(5)** |
| --- | --- | --- | --- | --- | --- |
| **PMC fiscal reform** | 0.613* | 0.992*** | 0.690* | -0.804*** | 0.140 |
|  | (0.348) | (0.381) | (0.360) | (0.294) | (0.153) |
| **Year fixed effect** | Yes | Yes | Yes | Yes | Yes |
| **County fixed effect** | Yes | Yes | Yes | Yes | Yes |
| **Control variables** | Yes | Yes | Yes | Yes | Yes |
| **8 key determinants ×Year** | Yes | Yes | Yes | Yes | Yes |
| **Treatment trend** | Yes | Yes | Yes | Yes | Yes |
| **Year coverage** | 2000-2013 | 2000-2013 | 2000-2013 | 2000-2013 | 2000-2013 |
| **Adjusted R-squared** | 0.314 | 0.400 | 0.345 | 0.215 | 0.233 |
| **Obs.** | 12764 | 12764 | 12764 | 12764 | 12764 |

Notes: The table presents the estimates of the impact of the PMC fiscal reform on the tax burden to firms. In columns (1)-(3), the dependent variables are tax burden in zombie firms, zombie SOEs, and zombie NSOEs, respectively. The identification method of zombie firms is the Nie method. In columns (4)-(5), the dependent variables are tax burden in newly added firms and other firms (firms excluding zombie firms and newly added firms), respectively. All observations are at the county-year level. *, **, and *** indicate statistical significance at the 10%, 5% and 1% levels respectively.

### Impacts of the PMC fiscal reform on the tax burden of firms by using the FN-CHK method

|  | **(1)** | **(2)** | **(3)** | **(4)** | **(5)** |
| --- | --- | --- | --- | --- | --- |
| **PMC fiscal reform** | 0.503* | 0.607* | 0.791** | -0.804*** | 0.111 |
|  | (0.290) | (0.357) | (0.324) | (0.294) | (0.162) |
| **Year fixed effect** | Yes | Yes | Yes | Yes | Yes |
| **County fixed effect** | Yes | Yes | Yes | Yes | Yes |
| **Control variables** | Yes | Yes | Yes | Yes | Yes |
| **8 key determinants ×Year** | Yes | Yes | Yes | Yes | Yes |
| **Treatment trend** | Yes | Yes | Yes | Yes | Yes |
| **Year coverage** | 2000-2013 | 2000-2013 | 2000-2013 | 2000-2013 | 2000-2013 |
| **Adjusted R-squared** | 0.226 | 0.336 | 0.312 | 0.215 | 0.211 |
| **Obs.** | 12764 | 12764 | 12764 | 12764 | 12764 |

Notes: The table presents the estimates of the impact of the PMC fiscal reform on the tax burden to firms. In columns (1)-(3), the dependent variables are tax burden in zombie firms, zombie SOEs, and zombie NSOEs, respectively. The identification method of zombie firms is the Nie method. In columns (4)-(5), the dependent variables are tax burden in newly added firms and other firms (firms excluding zombie firms and newly added firms), respectively. All observations are at the county-year level. *, **, and *** indicate statistical significance at the 10%, 5% and 1% levels respectively.

### Impacts of the PMC fiscal reform on government financial support by using the Tan method

|  | **(1)** | **(2)** | **(3)** | **(4)** | **(5)** |
| --- | --- | --- | --- | --- | --- |
| **PMC fiscal reform** | 0.467* | 0.842*** | 0.145 | -0.426* | 0.018 |
|  | (0.265) | (0.303) | (0.265) | (0.244) | (0.095) |
| **Year fixed effect** | Yes | Yes | Yes | Yes | Yes |
| **County fixed effect** | Yes | Yes | Yes | Yes | Yes |
| **Control variables** | Yes | Yes | Yes | Yes | Yes |
| **8 key determinants ×Year** | Yes | Yes | Yes | Yes | Yes |
| **Treatment trend** | Yes | Yes | Yes | Yes | Yes |
| **Year coverage** | 2000-2013 | 2000-2013 | 2000-2013 | 2000-2013 | 2000-2013 |
| **Adjusted R-squared** | 0.249 | 0.301 | 0.307 | 0.195 | 0.194 |
| **Obs.** | 12764 | 12764 | 12764 | 12764 | 12764 |

Notes: The table presents the estimates of the impact of the PMC fiscal reform on the government financial support to firms. In columns (1)-(3), the dependent variables are the government’s financial support to zombie firms, zombie SOEs, and zombie NSOEs. The identification method of zombie firms is the Nie method. In columns (4)-(5), the dependent variables are the government’s financial support to newly added firms and other firms (firms excluding zombie firms and newly added firms), respectively. All observations are at the county-year level. *, **, and *** indicate statistical significance at the 10%, 5% and 1% levels respectively.

### Impacts of the PMC fiscal reform on government financial support by using the FN-CHK method

|  | **(1)** | **(2)** | **(3)** | **(4)** | **(5)** |
| --- | --- | --- | --- | --- | --- |
| **PMC fiscal reform** | 0.460** | 0.779*** | 0.339 | -0.426* | -0.026 |
|  | (0.203) | (0.276) | (0.251) | (0.244) | (0.103) |
| **Year fixed effect** | Yes | Yes | Yes | Yes | Yes |
| **County fixed effect** | Yes | Yes | Yes | Yes | Yes |
| **Control variables** | Yes | Yes | Yes | Yes | Yes |
| **8 key determinants ×Year** | Yes | Yes | Yes | Yes | Yes |
| **Treatment trend** | Yes | Yes | Yes | Yes | Yes |
| **Year coverage** | 2000-2013 | 2000-2013 | 2000-2013 | 2000-2013 | 2000-2013 |
| **Adjusted R-squared** | 0.195 | 0.255 | 0.256 | 0.195 | 0.183 |
| **Obs.** | 12764 | 12764 | 12764 | 12764 | 12764 |

Notes: The table presents the estimates of the impact of the PMC fiscal reform on the government financial support to firms. In columns (1)-(3), the dependent variables are the government’s financial support to zombie firms, zombie SOEs, and zombie NSOEs, respectively. The identification method of zombie firms is the Nie method. In columns (4)-(5), the dependent variables are the government’s financial support to newly added firms and other firms (firms excluding zombie firms and newly added firms), respectively. All observations are at the county-year level. *, **, and *** indicate statistical significance at the 10%, 5% and 1% levels respectively.

### References in Appendix

1. Liu Y, Alm J. “Province-Managing-County” fiscal reform, land expansion, and urban growth in China. Journal of Housing Economics. 2016;33: 82–100. doi:10.1016/j.jhe.2016.05.002

2. Jia J, Ding S, Liu Y. Decentralization, incentives, and local tax enforcement. Journal of Urban Economics. 2020;115: 103225. doi:10.1016/j.jue.2019.103225

3. Huang B, Gao M, Xu C, Zhu Y. The impact of Province-Managing-County fiscal reform on primary education in China. China Economic Review. 2017;45: 45–61. doi:10.1016/j.chieco.2017.06.001

4. Li P, Lu Y, Wang J. Does flattening government improve economic performance? Evidence from China. Journal of Development Economics. 2016;123: 18–37. doi:10.1016/j.jdeveco.2016.07.002

5. Caballero RJ, Hoshi T, Kashyap AK. Zombie Lending and Depressed Restructuring in Japan. American Economic Review. 2008;98: 1943–1977. doi:10.1257/aer.98.5.1943

6. Huang Z, Du X. Holding the market under the stimulus plan: Local government financing vehicles’ land purchasing behavior in China. China Economic Review. 2018;50: 85–100. doi:10.1016/j.chieco.2018.04.004

7. Dang D, Fang H, He M. Economic policy uncertainty, tax quotas and corporate tax burden: Evidence from China. China Economic Review. 2019;56: 101303. doi:10.1016/j.chieco.2019.101303

8. Wu GL, Feng Q, Li P. Does local governments’ budget deficit push up housing prices in China? China Economic Review. 2015;35: 183–196. doi:10.1016/j.chieco.2014.08.007

9. Shen G, Chen B. Zombie firms and over-capacity in Chinese manufacturing. China Economic Review. 2017;44: 327–342. doi:10.1016/j.chieco.2017.05.008

10. Brandt L, Van Biesebroeck J, Wang L, Zhang Y. WTO Accession and Performance of Chinese Manufacturing Firms. American Economic Review. 2017;107: 2784–2820. doi:10.1257/aer.20121266
